# Building a Bridge Between Ambient MS and LC‐MS by Non‐Exhaustive Microdesorption

**DOI:** 10.1002/anie.202504080

**Published:** 2025-07-22

**Authors:** Wei Zhou, Janusz Pawliszyn

**Affiliations:** ^1^ Department of Chemistry University of Waterloo Waterloo Ontario N2L 3G1 Canada

**Keywords:** Ambient mass spectrometry, Anti‐doping, Rapid screening, Sequential analysis, Solid‐phase microextraction

## Abstract

Ambient mass spectrometry (AMS) offers rapid screening but faces challenges in analyzing complex samples due to high matrix effects. The absence of a separation step can also lead to false positives due to the isomers or isobars. In this study, a sequential analysis strategy which combines ambient MS and LC‐MS based on the non‐exhaustive microdesorption in solid‐phase microextraction (SPME) was developed for the first time. By combining coated blade spray (CBS)‐MS with LC‐MS, in the first step, a few microliters of solvent were used for non‐exhaustive desorption with high enrichment factor for rapid screening by CBS‐MS. For the suspicious samples, the remaining analytes on the SPME coating undergo exhaustive desorption, then followed by LC‐MS confirmation. The matrix‐compatible coating used in the SPME device significantly reduces matrix effects while enhancing sensitivity through analyte enrichment. This method is environmentally friendly, utilizing only a few microliters of organic solvents for screening. The approach was rigorously validated, both theoretically and experimentally, and successfully applied to anti‐doping testing, enabling detection of 53 prohibited substances in urine samples by integrating CBS‐MS with LC‐MS.

## Graphical abstract

A sequential analysis strategy was developed to integrate rapid ambient mass spectrometry (MS) screening with LC‐MS confirmation using a single solid‐phase microextraction (SPME) device. The approach involves an initial non‐exhaustive microdesorption for ambient MS screening, followed by a second desorption for LC‐MS confirmation of only the disputed samples. This workflow enhances throughput while maintaining sensitivity and minimizing matrix effects. The method has been applied for high throughput anti‐doping test.

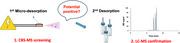

## Introduction

High throughput and rapid screening mass spectrometry (MS) methods have revolutionized analytical sciences, offering swift and efficient analysis of numerous samples.^[^ ^1^ ^]^ These methods offer unparalleled sensitivity and specificity, allowing for the detection and quantification of trace compounds in complex matrices.^[^ ^2^ ^]^ Their versatility and automation capabilities make them indispensable in many fields, including drug discovery,^[^ ^3^, ^4^, ^5^ ^]^ food safety,^[^ ^6^, ^7^ ^]^ environmental monitoring,^[^ ^8^, ^9^ ^]^ and anti‐doping tests.^[^ ^10^, ^11^ ^]^

Ambient mass spectrometry (AMS), employing ambient ionization techniques^[^ ^12^ ^]^ such as desorption electrospray ionization (DESI),^[^ ^13^ ^]^ direct analysis in real time (DART),^[^ ^14^ ^]^ and paper spray ionization (PSI),^[^ ^15^ ^]^ enables rapid screening without the need for extensive sample preparation or time‐consuming chromatographic separation.^[^ ^16^, ^17^ ^]^ However, AMS encounters challenges when detecting trace amounts of analytes in complex samples, such as biofluids and tissue, due to matrix effects and interferences that compromise sensitivity through ion suppression and increased background noise.^[^ ^18^, ^19^, ^20^ ^]^ To improve the performance for analysis of complex samples, coupling simple, high throughput sample preparation techniques presents an effective solution to these challenges.^[^ ^21^, ^22^ ^]^ Solid‐phase microextraction (SPME) is a versatile sample‐preparation technique based on the extraction of analytes using solid or semi‐solid coating materials. It integrates sampling, extraction, enrichment, and cleanup into a single step.^[^ ^23^ ^]^ Advances in SPME, including matrix‐compatible coating materials and high throughput extraction systems, provide viable solutions for these applications. The matrix‐compatible coating materials, composing of high‐capacity sorbent particles embedded in polymeric binders like polyacrylonitrile (PAN),^[^ ^24^ ^]^ not only enhance analyte enrichment, but also perform a clean‐up function, enabling selective extraction of small molecules while excluding larger matrix components using the pores in the binder. Moreover, the adoption of high‐throughput and automated SPME systems can process up to hundreds of samples simultaneously using well plate and significantly reduce sample preparation times to just a few seconds per sample,^[^ ^25^ ^]^ thereby maintaining overall method throughput without compromising efficiency.

In the past years, AMS techniques have been coupled with advanced MS instrumentations, featuring high selectivity, scan speed and sensitivity. For example, DESI, DART and PSI have been integrated with the high‐resolution MS (HRMS) for applications such as anti‐doping and illicit drugs testing in urine and fingerprint, achieving improved selectivity.^[^ ^26^, ^27^, ^28^ ^]^ PSI has also been coupled with ion mobility‐MS/MS for the strain‐level discrimination of bacteria, achieving impressive prediction rates up to 62.5% and 73.5% in the negative and positive ion modes, respectively.^[^ ^29^ ^]^ These advanced MS detection methods, including tandem MS, HRMS, ion mobility MS and MSn by ion trap MS, enhance the selectivity and the results align well with traditional sample preparation‐chromatographic separation (LC/GC)‐MS working flow, especially when targeting a small number of targeted analytes with detailed optimization. Despite these advancements, AMS is still recognized as a rapid screening tool rather than a standalone analytical method in practical applications. As in many cases, such as anti‐doping, illicit drugs, and pesticides screening, large number of targeted analytes must be simultaneously screened, often within varied composition of sample matrixes. Isomers and isobars can exhibit identical MS patterns to targeted analytes, impacting the accuracy of test results, and/or increasing the background noise, causing false positives.^[^ ^26^, ^30^ ^]^ Although ion mobility MS can provide quick separation in some cases,^[^ ^31^ ^]^ it is difficult to optimize a specific parameter that all potential isomers or isobars can be separated. For instance, in anti‐doping tests mandated by the World Anti‐Doping Agency's (WADA), more than 300 small molecules, including several groups of isomers like phentermine, 2‐methylamphetamine, and 4‐methylamphetamine, or synephrine and phenylephrine, share the same m/z and MRM transitions with similar collision cross‐section (CCS) values.^[^ ^32^ ^]^ For above reasons, traditional workflows including sample preparation, LC or GC separation followed by MS detection are the golden standard in many regulations. The best approach would be to use AMS for rapid screening, with the disputed cases re‐analyzed again by sample preparation‐LC/GC‐MS for confirmation. However, re‐analyzing these disputed samples from scratch requires additional sample aliquots and decreases the overall throughput of the method. It is also impractical in scenarios such as on‐site sampling or in vivo analysis, where additional samples or sampling opportunities may not be readily available. Developing technology which could combine these two approaches would simplify the workflow and provide the opportunity to eventually promote AMS included in official technical guidelines.

In this study, we developed a sequential analysis strategy that integrates rapid screening by AMS with confirmation by LC‐MS using a single SPME sampling. By employing coated blade spray‐MS (CBS‐MS) in the first step, a few microliters of the solvent were applied to partially desorb the analytes from the extraction phase, providing a high enrichment factor with increased sensitivity. Due to the non‐exhaustive feature of microdesorption, the remaining analytes in the coating material can undergo a second, exhaustive desorption step, followed by LC‐MS analysis for the suspected samples. This approach allows rapid screening and confirmation using a single SPME device, with sampling and sample preparation conducted only once. Our research began with a fundamental study of the micro‐desorption process, demonstrating that it can increase method sensitivity while remaining more analytes in the coating material, and both analysis processes are quantitative. The theoretical results were experimentally demonstrated by coupling CBS‐MS and LC‐MS analysis. This is the first time micro‐desorption has been studied in detail, and considered as the bridge to connect AMS with LC‐MS. Finally, we developed a high‐throughput, semi‐automated sequential analysis protocol combining CBS‐MS and LC‐MS, specifically tailored for anti‐doping application.

## Results and Discussion

### Study Micro‐desorption in SPME

SPME device is composed of small amounts of extraction phase bonded to solid support. While the non‐exhaustive extraction theory of SPME has been extensively studied,^[^ ^33^ ^]^ few studies have investigated the desorption process. For the liquid desorption using organic solvents, we can consider the coating as the sample, and the desorption solution as the extraction phase. In this case, the equilibrium state can be described using the same principles as the extraction process, where the following equation applies at equilibrium:

(1) $$C_{0}V_{c}=C_{c}V_{c}+C_{d}V_{d}$$

where *C*_0_ is the initial concentration of the analyte in the coating, *C_c_* is the equilibrium concentration in the coating, *C_d_* is the equilibrium concentration in the desorption solution, and *V_c_* and *V_d_* denote the volumes of the coating and desorption solution, respectively. The distribution coefficient *K_dc_* of the analyte between the desorption solution and coating is defined as:

(2) $$K_{dc}=\frac{C_{d}}{C_{c}}\;$$

Equations (1) and (2) can be combined and rearranged into:

(3) $$C_{d}=C_{0}\;\frac{K_{dc}V_{c}}{K_{dc}V_{d}+V_{c}}$$

When we want to consider the influence of *V_d_* on *C_d_*, and define *C*_0_, *K_dc_* and *V_c_* as certain values, which are all > 0, this equation can be simplified to:

(4) $$y=\frac{a}{\frac{a}{b}x+1}\;\times C_{0}$$

where *a* represents *K_dc_*, *b* represents *V_c_*, *y* represents *C_d_*, and *x* represents *V_d_*. As *a*, *b*, *x* and *C*_0_ are > 0, it is clear that *y* increases with the decrease of *x*. This means the concentration of the analyte in the desorption solution *C_d_* increases with the decrease of desorption volume *V_d_*.

The number of moles of analytes *n* desorbed in the solvent can be calculated by:

(5) $$n=C_{d}V_{d}=C_{0}\frac{K_{dc}V_{c}V_{d}}{K_{dc}V_{d}+V_{c}}$$

Which can be simplified to:

(6) $$y=\frac{abx}{ax+b}\;\times \;C_{0}=\frac{ab}{a+\frac{b}{x}}\;\times C_{0}$$

In this case, as *x* increases, *y* also increases, indicating that the amount of analyte in the desorption solution increases with an increase in desorption volume.

Equations (4) and (6) suggest that during SPME desorption, the concentration of the analytes in desorption solution increases with the decrease of the volume of desorption solution, while the amount of analyte in the desorption solution decreases when the desorption volume decreases. Equation (5) further indicates that the desorbed analyte *n* is linearly proportional to the amount of analyte in the coating *C*_0_, and that *C*_0_ is linearly proportional to the original concentration of analyte in the sample according to the previous study on the SPME extraction process.^[^ ^33^ ^]^ This demonstrates that the detection of the analyte in the desorption solution is quantitative, even when the analyte is non‐exhaustively desorbed using a small volume of desorption solution.

If a second desorption is applied after the first non‐exhaustive desorption, the amount of analyte in the second desorption solution, *n*_2_, can be defined as:

(7) $$\begin{array}{ccc} n_{2} & = & C_{0}\left(1-\frac{K_{\mathrm{dc}}V_{d1}}{K_{\mathrm{dc}}V_{d1}+V_{c}}\right)\;\;\frac{K_{\mathrm{dc}}V_{c}V_{d2}}{K_{\mathrm{dc}}V_{d2}+V_{c}}\\ & = & C_{0}\left(1-\frac{K_{\mathrm{dc}}V_{d1}}{K_{\mathrm{dc}}V_{d1}+V_{c}}\right)\frac{K_{\mathrm{dc}}V_{c}}{K_{\mathrm{dc}}+\frac{V_{c}}{V_{d2}}} \end{array}$$

where *V*_*d*1_ is the volume of the first desorption solution and *V*_*d*2_ is the volume of the second desorption solution. This equation suggests that the detection of the analyte in the second desorption solution is also quantitative, as *n*_2_ is also linearly proportional to *C*_0_. When *V*_*d*1_ is small for non‐exhaustive desorption, and *V*_*d*2_ is very large for exhaustive desorption, Equation (7) can be converted to:

(8) $$n_{2}=C_{0}\left(1-\frac{K_{\mathrm{dc}}V_{d1}}{K_{\mathrm{dc}}V_{d1}+V_{c}}\right)\;\;V_{c}=C_{0}V_{c}-C_{0}\frac{K_{\mathrm{dc}}V_{d1}V_{c}}{K_{\mathrm{dc}}V_{d1}+V_{c}}$$

From the above discussion, we can also conclude that the enrichment factor (E) in SPME is influenced not only by the *K_es_* during the extraction process, but also by the *K_dc_* and desorption volume (*V_d_*) during the desorption process.

**Figure 1:**
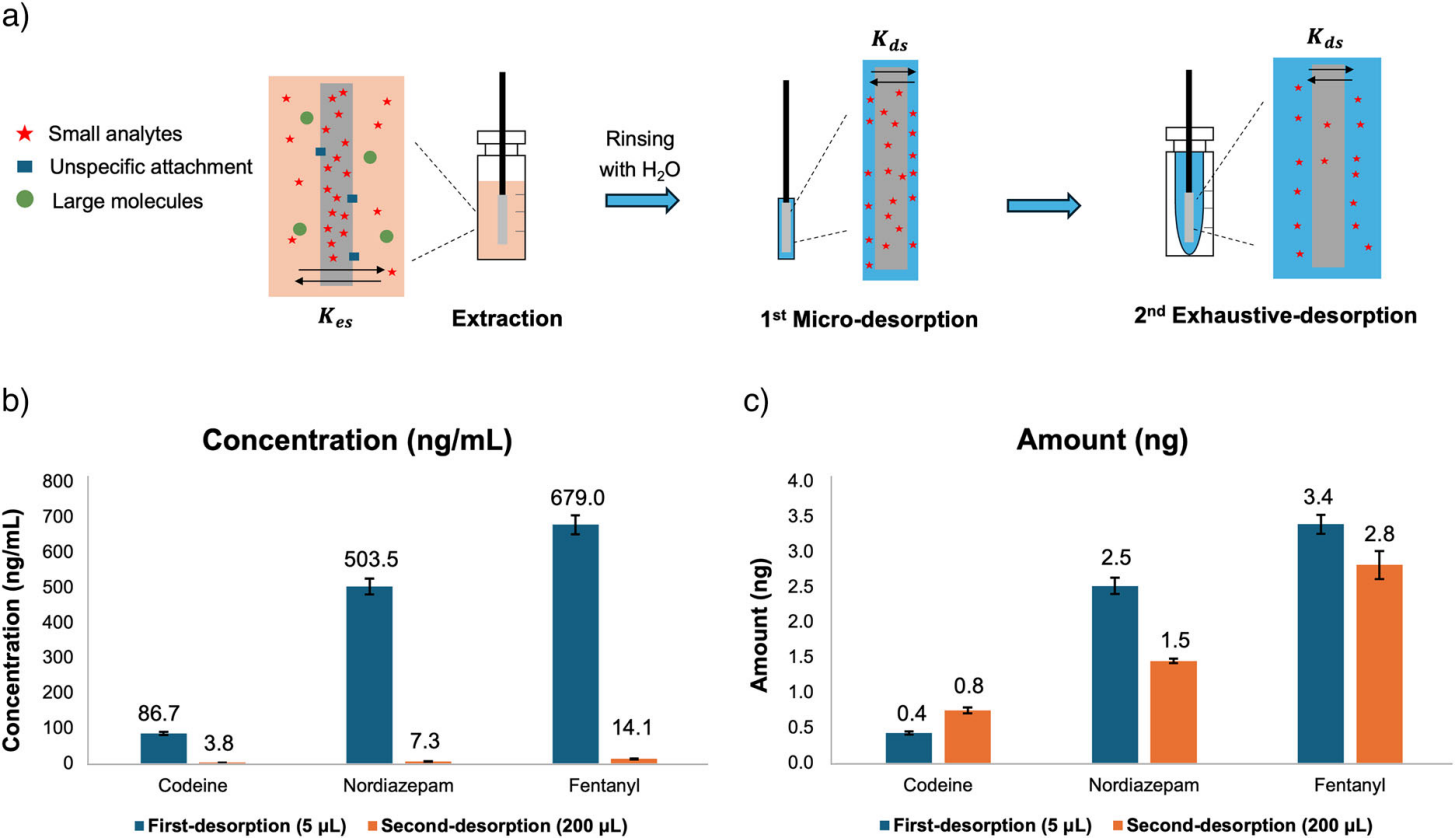
a) Schematic of SPME extraction, followed by micro‐desorption and second exhaustive desorption. After extraction, the SPME devices were quickly rinsing with water for 10 s to remove unspecific attachment on the surface. b) Concentration of analytes in the first desorption solution compared to the second desorption solution. c) Amount of analytes in the first desorption solution compared to the second desorption solution. Analytes were spiked in PBS solution at a concentration of 10 ng mL^−1^. Data points and error bars represent mean values ± standard deviation (SD) of four technical replicates (*n* = 4).

These mathematical relationships were experimentally validated through the extraction of three analytes—codeine (log P 1.2), nordiazepam (log P 2.9), and fentanyl (log P 4.1)—using SPME fibers with C18/PAN (Figure 1a). After extraction and quickly rinsing with H_2_O, the first non‐exhaustive desorption was conducted by immersing the fiber in a micro glass capillary containing 5 µL of ACN/H_2_O 8/2 (v/v) for 30 s. Then SPME fiber was then removed and subjected to a second desorption in 200 µL of ACN/H_2_O 8/2 (v/v) for 3 min. To prepare the first desorption solution for LC‐MS analysis, the 5 µL volume was further diluted with 55 µL of ACN/H_2_O 8/2 (v/v). The concentration levels (ng mL^−1^) and amounts (ng) of analytes in these two sequential desorption solutions were determined using instrument calibration curves and are presented Figure 1b and c. Both desorption steps demonstrated good reproducibility across three fibers (RSD% ≤ 7%). When using 5 µL of desorption solution, the concentrations of the analytes were significantly higher than those observed in the second desorption with 200 µL of the desorption solution (Figure 1b). However, the total amount of analytes in the two desorption solutions remained comparable (Figure 1c). This finding suggests that employing a small volume of desorption solution can achieve a high enrichment factor, yielding high analyte concentrations. It also confirms that desorption with a small volume, such as 5 µL of desorption solution, is non‐exhaustive, leaving a portion of the analytes in the coating for subsequent desorption.

At first glance, understanding the fundamentals of micro‐desorption may appear less relevant when using liquid chromatography (LC) for analysis, given that injection needles in LC samplers are unable to accommodate the small volumes involved. Typically, an LC system requires more than 100 µL of solution into the LC vial. Additionally, for most analytes, focusing occurs at the front of the column before solvent‐programmed separation, which reduces the need for highly concentrated small volumes. These are the reasons that micro‐desorption has not been investigated in detail. However, micro‐desorption becomes highly practical when SPME is directly coupled with MS, for example, in SPME‐nanoESI‐MS,^[^ ^34^ ^]^ SPME‐MOI‐MS,^[^ ^11^ ^]^ and CBS‐MS.^[^ ^35^ ^]^ As in these technologies, several microliters of desorption solution are used to achieve non‐exhaustive desorption and high enrichment.

CBS‐MS combines SPME with substrate‐ESI as an integrated technique for rapid analysis of complex samples.^[^ ^36^ ^]^ As illustrated in Figure S1a and S1b, the latest CBS design features a stainless‐steel blade coated with a matrix‐compatible coating layer on both sides. Previous researches have demonstrated that the CBS devices with biocompatibility coating can be directly applied for the both qualitative and quantitative analysis in complex samples such as whole blood, honey and even tissue with promising analytical performance.^[^ ^37^, ^38^, ^39^ ^]^ Compared to other substrate‐ESI‐based ambient techniques, such as paper spray, probe ESI (PESI), or wooden tip spray, the highly reproducible arc‐shaped tip of the CBS blade facilitates highly reproducible ESI spraying.^[^ ^40^ ^]^ Further, the wide MS detection window in CBS‐MS allows for simultaneous screening of hundreds of analytes via multiple reaction monitoring (MRM) transitions. To further increase the throughput of the method, an automated and high throughput SPME system^[^ ^41^ ^]^ (Figure S1c and S1d) capable of processing up to 96 samples simultaneously can be employed to achieve average sample preparation times as low as 26 s per sample.

### Coupling CBS‐MS with LC‐MS for Anti‐doping Application

**Figure 2:**
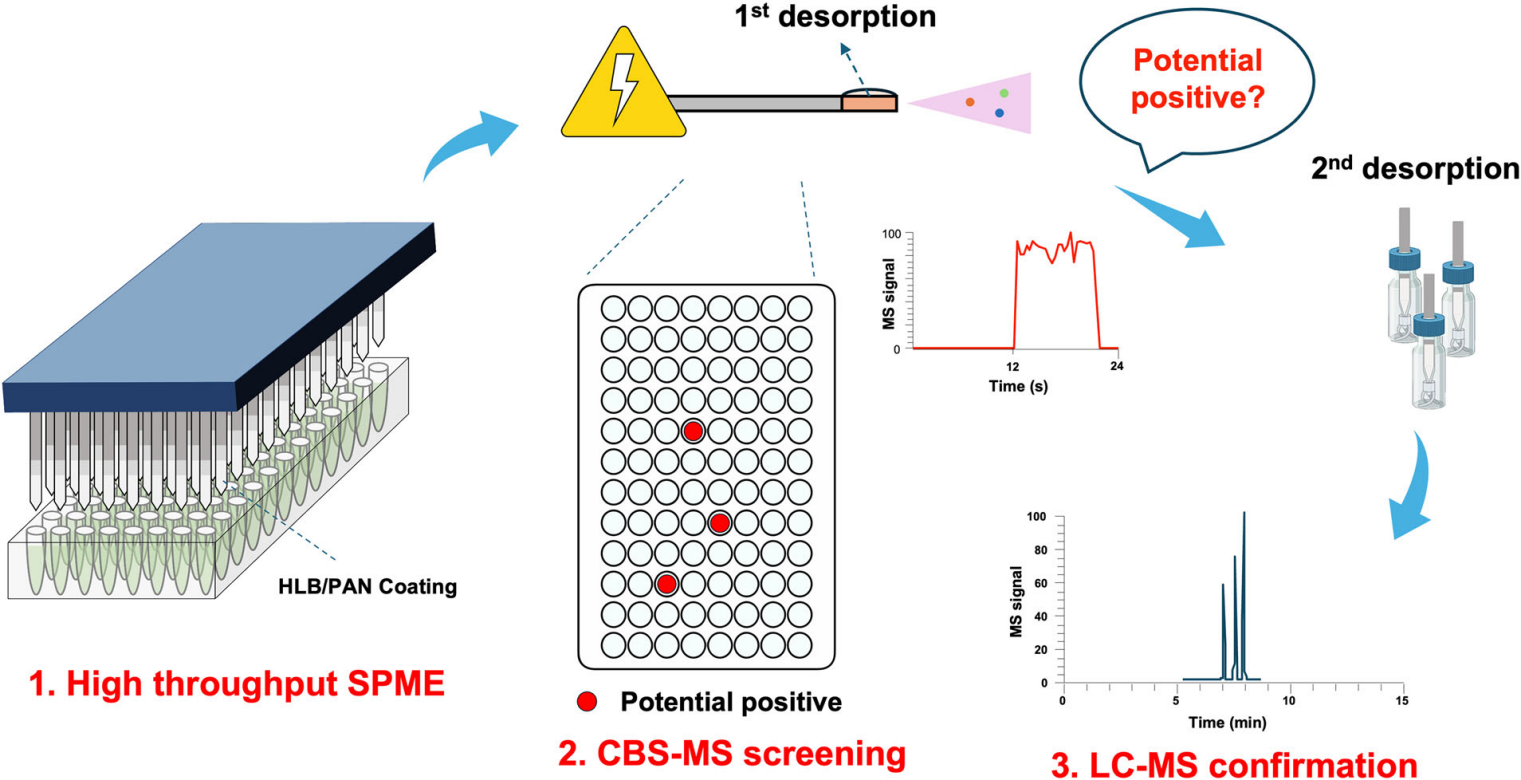
Schematic of the sequential analysis approach for anti‐doping applications. CBS‐MS with micro‐desorption is used for rapid screening, and SPME blades from potential positive cases undergo a second desorption in the LC vials followed by LC‐MS confirmation.

During large sporting events, such as the Olympic games, rapid analysis of a substantial number of samples within a limited timeframe is critical. For instance, at the Beijing Winter Olympic Games, 3165 samples were analysed, necessitating significant resources, including over 100 anti‐doping experts and 26 LC/GC‐MS instruments.^[^ ^42^ ^]^ To meet these demands, participating laboratories often have to operate 24 h a day. Given the constraints faced by local laboratories, and the large number of athletes that compete in such sporting events, increasing the throughput of anti‐doping analytical protocols is essential. Additionally, automation of the sample preparation and analytical workflows is crucial, as it reduces dependence on the expertise of individual researchers while also enhancing the reproducibility and reliability of the method.^[^ ^43^ ^]^ Given the previously discussed advantages of AMS, these technologies have emerged as a desirable alternative for World Anti‐Doping Agency (WADA) applications. However, when applied for anti‐doping, hundreds of the small molecular prohibited substances are required to be screened simultaneously. As mentioned in the Introduction section, potential false‐positive causes could happen because of the isomers or isobars either from listed prohibited substances or the sample matrix. In such case, confirmation by LC‐MS directly for suspected samples is necessary. In this work, using the “bridge” of micro‐desorption in SPME, a sequential analysis strategy (Figure 2) is proposed that enables high throughput and rapid screening with CBS‐MS, followed by the confirmation step by LC‐MS for the disputed samples using the same SPME device. Using this strategy, comprehensive sample preparation from the beginning using additional sample aliquots is not required.

**Figure 3:**
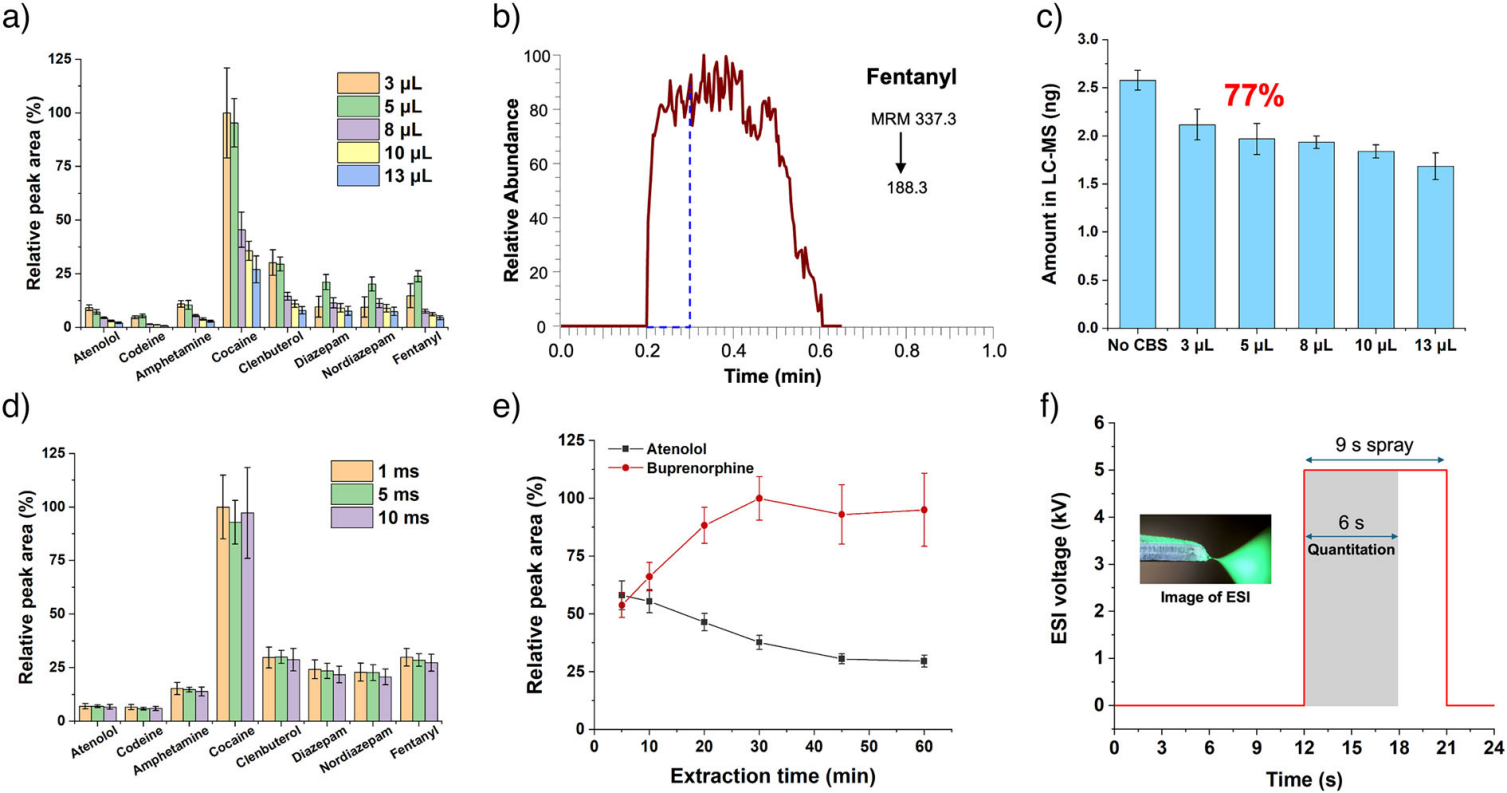
Optimization of the sequential analysis protocol. a) Optimization of the desorption solution volume in CBS‐MS. b) CBS‐MS chronogram of fentanyl with 5 µL desorption solution volume. c) Impact of the desorption solution volume used during the first CBS‐MS step on the amount of analyte attained via the second desorption solution, using fentanyl as an example. d) Optimization of dwell time. e) Extraction time profiles for atenolol and buprenorphine. f) Optimized high‐voltage protocol for CBS‐MS analysis. The inset shows an image of the ESI spray captured using a microscopic camera under green laser illumination. Data points and error bars in the method optimization figures (a) to (e) represent mean values ± standard deviation (SD) of four technical replicates (*n* = 4). Complete method optimization data are presented in Figures S2 and S3.

The CBS‐MS method was optimized using 16 drugs of abuse, representing a wide range of log *p* values. First, the desorption solution composition and desorption time were optimized, as shown in Figure S2a and S2b. To achieve better overall sensitivity and good reproducibility, a desorption solution composed of MeOH/ACN/H_2_O 85/10/5 (V/V/V) and a desorption time of 12 s were selected. The influence of desorption volume was also studied, with results shown in Figure 3a. As the desorption volume increased, the MS signal decreased, correlating with a lower enrichment factor. The use of 3 µL of desorption solution resulted in a high RSD% due to an unstable ESI spray. However, with 5 µL, a stable ESI spray of 18 s can be obtained (Figure 3b) with high sensitivity. As shown in Figure 3c, increasing the volume of the desorption solution in CBS‐MS also results in a decrease in the amounts of analyte attained via the second desorption with 200 µL of desorption solution. For instance, when using 5 µL of desorption solution in CBS, the amount of fentanyl in the sequential desorption solution was about 77% of that attained via direct desorption in 200 µL of solution without CBS, indicating that a significant portion of the analyte remained on the coating after the first non‐exhaustive desorption. For the other analytes (Table S1), this ratio ranged between 62% and 93%. The sequential desorption demonstrated good reproducibility with RSD% ≤ 16% (*n* =  4). In addition to the above discussed factors, it should be noted that the percentages of analytes remaining on the coating following the first desorption step are also dependent on the partition coefficients of individual analytes between the HLB/PAN coating and the desorption solution. Further, the significant amount of analytes observed to remain on the coating following micro‐desorption are also attributed to the CBS blade being coated on both sides, with only one side of the coating desorbed during the first desorption.

In triple quadrupole MS, high scanning speed ensures that more MRM transitions can be detected within a given detection window. Dwell times of 1, 5, and 10 ms (Figure 3d) were tested, with results suggesting that there were no significant differences between them. Thus, a dwell time of 1 ms was selected, allowing our MS instrument to perform 500 MRM scans per second. This means that in the 18 s detection window of CBS‐MS using 5 µL of desorption solution, 9000 MRM scan points can be achieved. If necessary, the detection window of CBS‐MS can be further extended by increasing the volume of the desorption solution. Extraction time was also optimized, as shown in Figure 5f. For non‐polar analytes, such as buprenorphine, the peak area increased up until 30 min due to the increasing amount of analyte being extracted over time. However, for polar compounds, such as atenolol, the peak area decreased with longer extraction times, likely due to achieving fast extraction equilibrium, then undergoing a displacement effect by non‐polar analytes entering the coating.^[^ ^44^ ^]^ Thus, considering both overall sensitivity and throughput, an extraction time of 20 min was selected. Full optimization data are shown in Figures S2 and S3.

The final CBS‐MS method setup is depicted in Figure 3f. After adding the desorption solution, the ESI voltage was kept at 0 kV for 12 s to desorb the analytes, followed by application of 5 kV on the blade for 9 s to generate the ESI spray. In our subsequent proof‐of‐concept anti‐doping application, only 53 analytes and 19 internal standards (IS) were analysed; given the high scanning speed used in this application, only the first 6 s were used for quantitation. After CBS‐MS analysis, the SPME blades underwent a second desorption step in 200 µL of the same desorption solution, which was subsequently submitted to LC‐MS/MS analysis. The representative MS chronograms of CBS‐MS and chromatograms from LC‐MS analysis are shown in Figures S4–S7. The log *p* values and MRM parameters of the prohibited substances are listed in Table S2.

**Figure 4:**
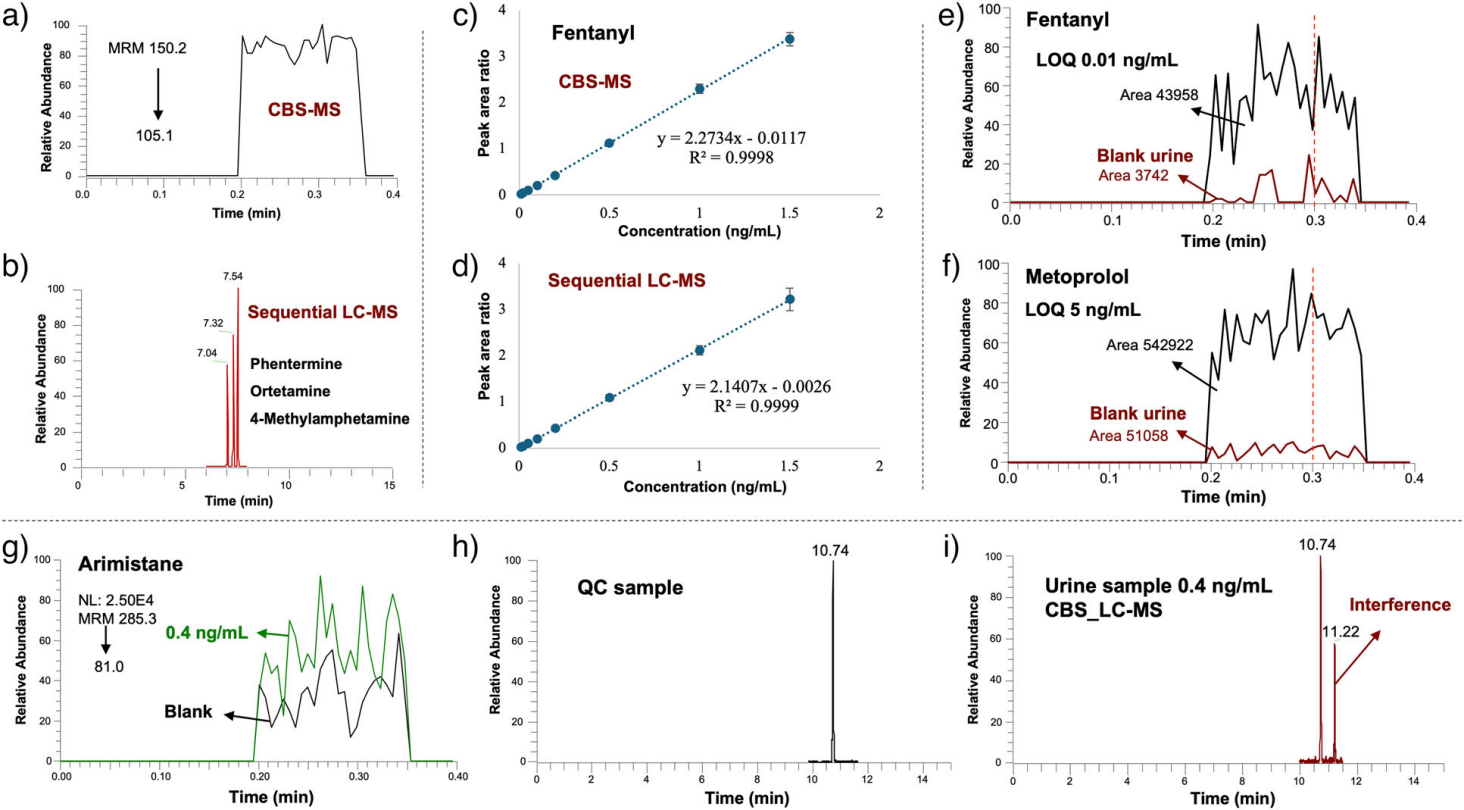
a) CBS‐MS chronogram and b) LC‐MS chromatogram of phentermine, ortetamine, and 4‐methylamphetamine. Due to their identical MRM transitions, the CBS‐MS chronogram represents a combined signal for all three compounds. c) Calibration curve of fentanyl obtained by CBS‐MS and by d) sequential LC‐MS following second desorption. Data points and error bars in c) and d) represent mean values ± standard deviation (SD) of four technical replicates (*n* = 4) at the calibration levels of 0, 1 and 1.5 ng mL^−1^, and represent mean values ± standard deviation (SD) of seven technical replicates (*n* = 7) at other calibration levels. e) CBS‐MS chronograms of blank and spiked urine samples at the limit of quantification (LOQ) level for fentanyl. f) CBS‐MS chronograms of blank and spiked urine samples at LOQ level for metoprolol. g) CBS‐MS chronograms of arimistane of blank and spiked urine samples at 0.4 ng mL^−1^. h) LC‐MS chromatograms for arimistane in instrument QC samples, and i) LC‐MS chromatograms for arimistane in extracted urine samples spiked with 0.4 ng mL^−1^ of arimistane, with arimistane peaks observed at 10.74 min and interference peak observed in 11.22 min.

The sequential analysis protocol, which integrates CBS‐MS with LC‐MS, was applied for the analysis of 53 representative small molecular analytes, belonging to 10 different classes of compounds listed as prohibited substances in the WADA list.^[^ ^45^ ^]^ As previously discussed, this method offers critical advantages, including the ability to distinguish isomers and mitigate potential false positives caused by sample matrix effects. This is achieved by subjecting SPME blades flagged as potential positives to a second desorption step followed by LC‐MS confirmation. For instance, phentermine, 2‐methylamphetamine and 4‐methylamphetamine are three prohibited isomers listed under S6 stimulants by WADA. The CBS‐MS results (Figure 4a) could not differentiate these analytes based on their MS signals alone, as they share identical MRM transitions. In such cases, the urine sample would be marked as a potential positive, and the corresponding SPME blade would undergo a second desorption for LC‐MS confirmation. Using a simple C18 column, these three analytes can be effectively separated (Figure 4b), thereby providing definite identification and confirmation.

The linearity and sensitivity of the method were evaluated, yielding promising quantitative results. As illustrated in Figure 4c and d, and detailed in Tables S3 and S4, both CBS‐MS and the sequential LC‐MS demonstrated good quantitative results, with linear correlation coefficients (*R* ^2^) ≥0.9934 for CBS‐MS, and *R* ^2^ ≥0.9868 for sequential LC‐MS. Sensitivity was assessed by defining the LOQs at the tested concentration levels with a signal‐to‐noise ratio (S/N) ≥10. For CBS‐MS, 45 of the 53 tested compounds met the minimum required performance level (MRPL) set by WADA (Table S3). In the case of sequential LC‐MS analysis, 50 analytes met the MPRL. However, three polar analytes—acetazolamide (log *p* ‐0.3), synephrine (log *p* ‐0.6) and phenylephrine (log *p* ‐0.3)—exhibited LOQs higher than their respective MRPL concentrations in sequential LC‐MS. This is attributable to the extraction mechanisms of the HLB/PAN coating being primarily dependent on hydrophobic interactions, resulting in low extraction efficiency for these polar analytes. A comparison with standalone SPME‐LC‐MS was conducted, with results shown in Table S5. For most analytes, the LOQs were consistent with those obtained from sequential LC‐MS, due to the majority of these analytes remaining on the blades after the initial non‐exhaustive desorption. Typically, CBS‐MS exhibits similar sensitivity to SPME‐LC‐MS.^[^ ^20^ ^]^

However, for five analytes—clenbuterol, desidustat, anastrozole, arimistane, and methylprednisolone—sequential LC‐MS met the MRPL while CBS‐MS did not. This discrepancy is attributed to the high background noise originating from either the urine samples or the analytical system, as CBS‐MS lacks a separation step. For example, as shown in Figure 4e and f, fentanyl, which exhibited a low background noise in blank urine (peak area of 3742), had identical LOQs of 0.01 ng mL^−1^ for CBS‐MS and sequential LC‐MS. In contrast, metoprolol, with high background signals in blank urine (peak area of 51 058), had a much higher LOQ (5 ng mL^−1^) in CBS‐MS compared to the sequential LC‐MS (0.5 ng mL^−1^). High background noise was consistently observed in CBS‐MS analysis for these five analytes. For instance, as shown in Figure 4g–i, an interference peak at the retention time of 11.22 min was consistently present when analysing human urine samples for arimistane using LC‐MS. This interference contributed to the elevated background noise and high LOQ observed for arimistane in CBS‐MS.

In terms of sensitivity and coverage, for the polar analytes, where both CBS‐MS and sequential LC‐MS cannot meet the MRPL, combining with a separate dilute‐and‐shoot (DnS) method should help. As currently, no single sample preparation technique can comprehensively cover every small molecular prohibited substance on the WADA list. For instance, during the 2021 Tokyo Summer Olympics, solid‐phase extraction (SPE)‐LC‐MS was combined with DnS‐LC‐MS,^[^ ^46^ ^]^ while in the 2022 Beijing Winter Olympics, liquid‐liquid extraction (LLE)‐LC‐MS was used alongside DnS‐LC‐MS to achieve broader analyte coverage.^[^ ^42^, ^47^ ^]^ Therefore, it is feasible to integrate our sequential analysis method with complementary approaches, such as DnS‐LC‐MS, to achieve the required analyte coverage. For analytes that exhibit high background noise in CBS‐MS, coupling with an ion trap mass spectrometer capable of MSn analysis presents a promising solution, as MS3 can offer improved selectivity over traditional MS2 approaches used in triple quadrupole instruments, enabling better differentiation targeted analytes and interferences.^[^ ^48^ ^]^ Additionally, integrating CBS with high‐resolution Q‐TOF or ion mobility–Q‐TOF systems—may help reduce background interference and improve analytical specificity.^[^ ^49^, ^50^ ^]^ These strategies represent exciting avenues for future development and optimization of the CBS‐MS platform.

**Figure 5:**
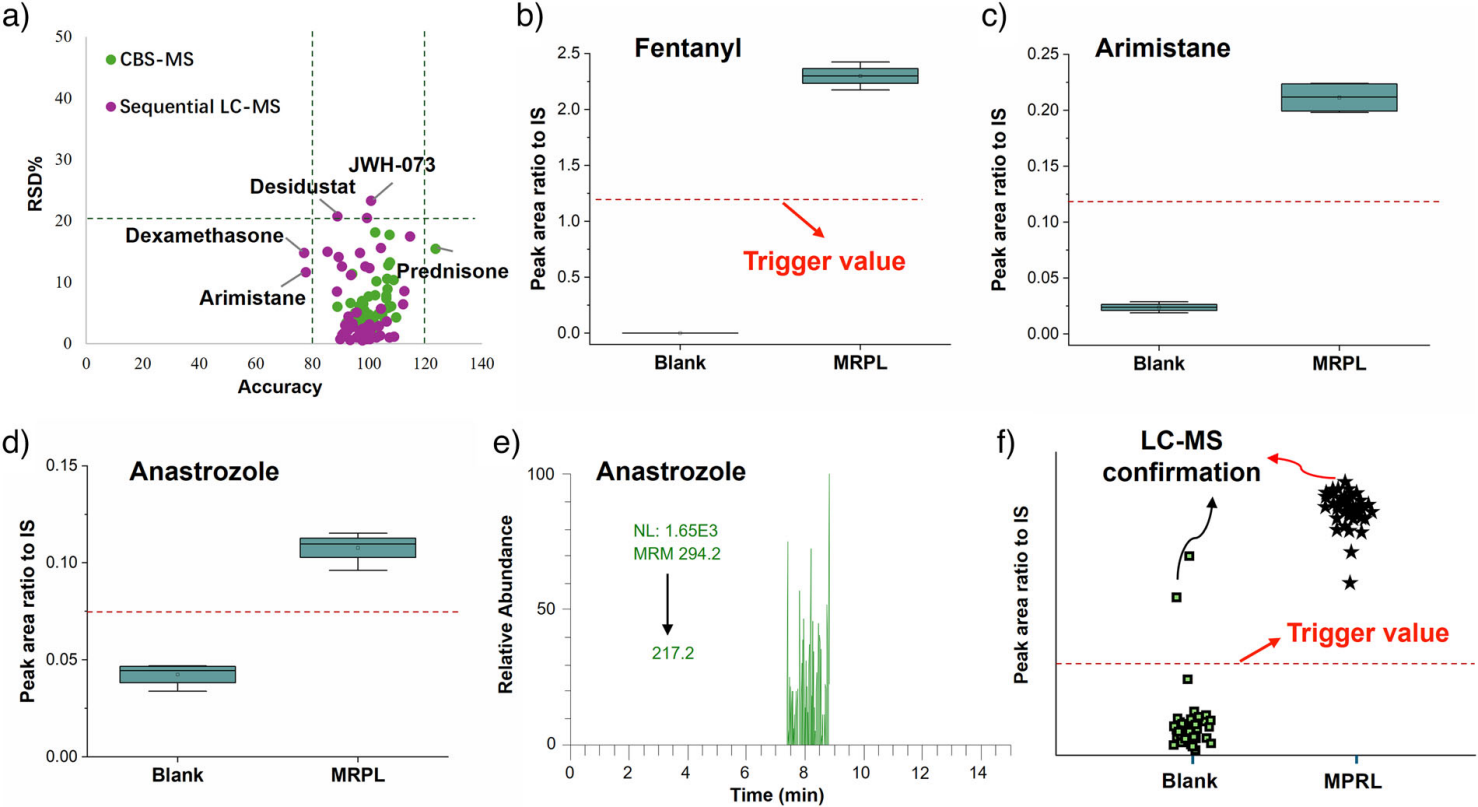
Validation results for CBS‐MS and sequential LC‐MS methods. a) Accuracy and RSD% for CBS‐MS and sequential LC‐MS. Data points represent mean values of seven technical replicates (*n* = 7). b–d) Box plots for fentanyl, arimistane, and anastrozole in blank urine blank and spiked urine samples at MRPL, with trigger values indicated by red dash lines. Data points in (b–d) represent four urine samples (*n* = 4). e) Chromatogram of anastrozole in blank urine No. 11 from sequential LC‐MS confirmation. f) Ideal trigger value set by WADA applications based on a larger sample set.

For method validation, accuracy was assessed by spiking blank urine samples at 3 concentration levels, including 25%, 50% and 100% MRPL. Recovery rates for CBS‐MS ranged from 84% to 124%, while those for sequential LC‐MS analysis ranged from 69% to 116% (Table S6). Reproducibility was evaluated by spiking urine samples at 50% of the MRPL, with seven replicates analysed on the same day and across 3 different days. As detailed in Table S7, the intra‐day RSDs% (*n* = 7) for CBS‐MS were ≤ 18% and inter‐day RSDs% (*n* = 3) were ≤ 16%. For sequential LC‐MS analysis, intra‐day RSDs% (*n* = 7) were ≤ 23%, and inter‐day RSDs% (*n* = 3) were ≤ 19%. As shown in Figure 5a, both CBS‐MS and sequential LC‐MS demonstrated promising accuracy and reproducibility, with most analytes achieving RSD% ≤ 20%, and accuracy ranging from 80% and 120% in both steps. The stability of the analytes in the desorption solution and on the blade was assessed by storing the blades and second desorption solutions after initial CBS‐MS analysis at room temperature (20 °C) for 12 h, followed by LC‐MS analysis. Considering the throughput of the initial CBS‐MS screening is approximately 1 min per sample, 12 h suffices for the first‐round screening of over 700 samples. The peak area ratios of prohibited substances to IS under these conditions were compared to those obtained from immediate analysis after the second desorption (Table S8). The results indicate that most analytes are stable in both the desorption solution and on the blade, with ratios ranging from 85% to 115% with RSD% of less than 20% (*n* = 5). There are two exceptions including desidustat and budesonide. These values for desidustat are 77% in the desorption solution and 61% on the blade, potentially due to the low sensitivity of this compound at a testing concentration of 1 ng mL^−1^, exactly at the limit of quantification (LOQ), and high RSD% values of 22% and 32% under these conditions, respectively. Budesonide displayed stability values of 76% in the desorption solution and 82% on the blade, suggesting instability of the compound under these conditions.

The selectivity of the method was evaluated using 12 blank urine samples from individuals who had not ingested any prohibited substances. Reliability was assessed by spiking these blank urine samples with the target analytes at MRPL. A trigger value for CBS‐MS screening was established to determine whether samples should be flagged as potential positives and subjected to sequential LC‐MS confirmation. As illustrated in Figure 5b, using fentanyl as an example, the trigger value was set as the average peak area ratio from blank urine samples (*n* = 4) and spiked samples at the MRPL concentration (*n* = 4), derived from calibration curves using mixed urine samples. Interestingly, for the five analytes where the CBS‐MS LOQs exceeded the MPRL, significant differences were still observed between blank urine and MRPL‐spiked samples (Figures 5c,d and S8). Selectivity and reliability for these five analytes were further tested. The ratio of the CBS‐MS screening results of the 12 blank samples and 12 spiked samples to the related trigger value were calculated and shown in Tables S9 and S10. If there are no false results in CBS‐MS screening, for the blank samples, these values are supposed to ≤ 1, and for spiked samples, these values are supposed to ≥1.

No false positives or negatives were observed in CBS‐MS screening for analytes meeting MPRL sensitivity requirements. However, false positive cases were identified for desidustat and anastrozole in 4 urine samples, and for methylprednisolone in three urine blank samples. False negatives were obtained for desidustat in one spiked urine sample, and anastrozole in four spiked urine samples. These samples were then subjected to sequential confirmation via a second desorption followed by LC‐MS analysis, where no false positives or negatives were observed (Figures 5e and S9). The results indicate that for analytes that meeting MRPL sensitivity, CBS‐MS screening is less prone to false positives or negatives. However, for analytes with lower sensitivity, despite observing significant differences in signals between blank and spiked samples during calibration, CBS‐MS screening has possibility for false results. These false results likely stem from matrix effects from urine samples, as the physical and chemical parameters of urine samples can vary between individuals or even between collections from the same individual at different times. As discussed earlier, reducing background noise through the use of HRMS or tandem MS could enhance the sensitivity of the method further, improving both selectivity and reliability. The results also demonstrated the advantage of our sequential analysis approach, as the LC‐MS confirmation process are less influenced by matrix effects, that provided reliable results with 0 false positive or negative cases for all the tested prohibited substances.

The trigger values for each prohibited substance were provisionally defined as part of a proof‐of‐concept experiment. Moving forward, these trigger values should be further refined through a comprehensive analysis of S/N ratios attained from a larger sample set, potentially including hundreds of urine samples from athletes. As illustrated in Figure 5f, the objective would be to establish trigger values at thresholds that effectively eliminating false negative outcomes, while also minimize the occurrence of false‐positive results in CBS‐MS, thereby reducing the number of samples requiring sequential LC‐MS confirmation. This refinement is crucial for ensuring both the efficiency and accuracy of the testing protocol in real‐world anti‐doping scenarios.

## Conclusion

In this work, we successfully developed a sequential analysis strategy integrating CBS‐MS with LC‐MS. The initial step employs online non‐exhaustive microdesorption with direct MS analysis, enabling rapid screening with high sensitivity because of the method's high enrichment factor. The non‐exhaustive nature of micro‐desorption permits remaining analytes in the SPME coating to undergo a second, exhaustive desorption, followed by confirmation via LC‐MS for disputed samples. This strategy has been studied both fundamentally and experimentally. As a proof‐of‐concept, it was applied to the anti‐doping analysis of 53 prohibited substances in urine samples, demonstrating that the method maintains the speed and efficiency of initial screening while ensuring the accuracy and reliability of confirmatory analyses. Despite limitations in the number of urine samples and available resources for getting more numbers of prohibited drug standards, the experiments showcased the potential of this method for future WADA studies involving larger datasets of athletes' urine samples and a broader spectrum of prohibited substances.

It is noteworthy that this paper describes a novel analytical strategy that is not restricted only to the CBS‐MS used in this paper but can be extended to different AMS techniques for rapid screening followed by a sequential confirmation step, as long as non‐exhaustive desorption happens in the initial phase. For example, this strategy can be used for DART‐MS if thin film SPME is utilized for sample enrichment, the thermal desorption via DART is non‐exhaustive. Different ionization techniques such as atmospheric pressure chemical ionization (APCI), direct analysis in real time (DART), dielectric barrier discharge ionization (DBDI) could be adopted to extend the range of detectable analytes. In addition, coupling CBS with more selective MS detection strategies—such as MSn analysis using ion trap MS or advanced ion mobility–MS platforms like cyclic Q‐TOF or timsTOF—could further mitigate background noise from co‐eluted interferences. These approaches have the potential to enhance the sensitivity and selectivity of the screening method for certain analytes.

While automation has been successfully integrated into the SPME process, manual intervention is still required for blade transfer, desorption, and subsequent AMS analysis. To advance this technology, we are collaborating with a commercial partner to develop a fully automated CBS‐MS system, which will incorporate a robotic arm capable of handling blade transfers, adding desorption solution, and applying high voltage. This fully automated system will further enhance the usability of this technology in industrial and clinical labs as well as government agencies. Moreover, this strategy has the potential to be used for on‐site analysis when coupled with miniature ion trap MS, such as for illicit drug testing at roadside checkpoints or music festivals, with potential positive cases either confirmed by MSn on‐site or forwarded to laboratories for second desorption and LC‐MS confirmation.

This approach also aligns with principles of green chemistry by minimizing solvent use—requiring only a few microliters of organic solvents for SPME‐MS screening when solvent desorption is used, and eliminating solvent use altogether if thermal desorption is employed. This alternative approach not only significantly reduces solvent waste but enhances throughput, making it both environmentally sustainable and efficient.

## Conflict of Interests

The authors declare no conflict of interest.

## Supporting information



Supporting Information

## Data Availability

The data that support the findings of this study are available from the corresponding author upon reasonable request.
