# Supplementary material for: Building a Bridge Between Ambient MS and LC‐MS by Non‐Exhaustive Microdesorption
Source: Angew Chem Int Ed Engl. 2025 Jul 22;64(34):e202504080. doi: 10.1002/anie.202504080 (PMC12363638; doi:10.1002/anie.202504080)
Supplement: Supplementary file 1 — Supporting Information [file ANIE-64-e202504080-s001.docx]

**SUPPORTING INFORMATION**

Building a Bridge Between Ambient MS and LC-MS by Non-Exhaustive Microdesorption

*Wei Zhou, Janusz Pawliszyn**

**TABLE OF CONTENTS**

**Methods**…………………………………………………………………………………………….**S2-S4**

**Figure S1-S9**……………………………………………………………….……………………….**S5-S8**

**Table S1-S10**………………………………………………………………..……………………….**S9-S25**

**Methods**

**Reagents and Materials.** LC-MS-grade methanol (MeOH), acetonitrile (ACN), and isopropanol (IPA) were purchased from Fisher Scientific (Hampton, NJ, USA). Formic acid (FA), ammonium formate (NH_4_HCOO), and the PBS tablets used to prepare the PBS solution (pH 7.4) were obtained from Sigma Aldrich (Oakville, ON, Canada). The standards and internal standards, as listed in Table S1 were purchased from Toronto Research Chemicals (North York, ON, Canada). All standards were dissolved in methanol or ACN at a concentration of 1 mg/mL, all internal standards were dissolved at a concentration of 0.1 mg/mL. A stock solution of standard mixture and internal standards mixture were prepared according to the MRPL of the substances and the extract MRPL concentration level of each analyte were listed in Table S1. Human urine was collected from healthy volunteers who had not taken any of the targeted prohibited substances. CBS blades with hydrophilic-lipophilic-balanced (HLB) particles (5 µm in diameter) and polyacrylonitrile (PAN) as binder were donated by Restek Corporation (Bellefonte, PA, USA). The coating length is 1 cm and thickness is 10 µm on each side. The photos of the commercial RESTEK blades are shown in Fig. S1a. The C18/PAN coated SPME fibers were donated by Millipore Sigma (Bellefonte, PA, USA). The coating thickness is 40 µm.

**Instruments and methods.** The TSQ Quantiva mass spectrometer from Thermo Scientific (San Jose, CA, USA) was used for the anti-doping application including CBS-MS and LC-MS. The CBS-MS interface, which consisted of an x-y-z stage and a blade holder, was constructed by the University of Waterloo Machine Shop. A Concept-96 system (Figure S1b) was used to create a high-throughput and automated SPME extraction process. It was co-developed with PAS Technology (Magdala, Germany). The holder for the CBS blades was fabricated by University of Waterloo Science Technical Services and was capable of holding 96 CBS blades and being used with the standard 96-well plates. Centrifuge concentrator from Eppendorf (Mississauga, ON, Canada) was used to dry the desorption solution before reconstitution.

For the CBS-MS, the blade was inserted into the holder, where high voltage was applied to it via a conductive stainless-steel rod. The position of the CBS blade could be easily adjusted by moving the x-y-z stage. In the optimum position, the blade was parallel with the inlet of the MS, which was situated 12 mm from the spray tip. In the optimum parameters, ESI voltage of 5 kV was selected for ESI spray in positive ionization mode. The sheath gas, aux gas and sweep gas were set to 0, and the ion transfer tube temperature was 325˚C. The Q1 and Q3 resolutions were 0.7. The spray time was 9 s, and MS signal in the first 6 s was used for quantitation. Dwell time of 1 ms was employed for each MRM transition. For the target analytes, two MRM transitions were used, one for quantitation and the other for qualification. For the internal standards, one MRM transition was used, and the detailed MS/MS information is listed in Table S1. The typical MS spectrum of the CBS-MS was shown in Fig. S2 and S3.

For the LC-MS, a Hypersil Gold C18 selectivity HPLC column (1.9 μm, 2.1 mm$\times$100 mm) from ThermoFisher Scientific (San Jose, CA, USA) was used for separation. The mobile phase A was water with 0.1% FA and the mobile phase B was methanol with 0.1% FA. The gradient started with 3% phase B and then increased to 90% until 9 min, and to 98% until 11 min, keep at 98% until 13 min, decreased to 3% until 13.2 min and then keep at 3% until 17 min. The injection volume was 10 μL. Column oven was 40˚C. For the MS, the spray voltage was 3.5 kV, sheath gas, aux gas and sweep gas were 50, 10 and 1 arb. Ion transfer tube temperature was 325˚C, vaporizer temperature was 350˚C. Q1 resolution was 0.7, Q3 resolution was 1.2, CID gas was 1.5 mTorr. The dwell time was 1 ms. The MS/MS parameters are the same as the CBS-MS method. The typical MS spectrum and LC-MS chromatograms of the targeted analytes were shown in Fig. S4 and S5.

For the experiments related to SPME fibers, Shimadzu LCMS 8060 (Kyoto, Japan) triple quadrupole mass spectrometer was used. For LC-MS, a Phenomenex (Torrance, CA, USA) Kinetex PFP column (2.1 mm*100 mm) with 1.7 μm particle was used. The flow rate was 300 μL/min. The mobile phase A was water with 0.1% formic acid and mobile phase B was ACN with 0.1% formic acid. The gradient was 10% B for 1.0 min, then linearly ramped to 100% B until 7.0 min, held there until 9.0 min, and then returned to 10% B at 9.2 min. This composition was kept until 11.0 min. The column oven temperature was set as 40 °C. Sample injection volume was 5 μL. For MS, the interface voltage was 4.0 kV in positive mode, the interface temperature was 300 °C, the desolvation line temperature was 250 °C, the heating block temperature was 400 °C, and the nebulizing gas, drying gas and heating gas flow were 3.0, 10.0 and 10.0 L/min, respectively. The collision gas pressure was 270 kPa with argon.

**Experiment protocols.** For the experiment using SPME fiber-LC-MS using micro glass capillary, the analytes were spiked at 10 ng/mL in 1.5 mL of PBS solution with 3 replicates. The coating length of the fibers is 5 mm. After extraction of 30 min using a benchtop shaker at 1500 rpm, the fibers were rinsed with H_2_O for 5 s, and then desorbed in 5 μL of ACN/H_2_O 8/2 (v/v) for 30 s. Then the SPME fiber was taken out for the second desorption in 200 µL of ACN/H_2_O 8/2 (v/v) for 3 min. 5 µL of the first desorption solution was diluted with 55 µL of the ACN/H_2_O 8/2 (v/v) to make it sufficient for LC-MS analysis. The concentration level (ng/mL) and the amount (ng) in these two sequential desorption solutions were calculated by instrument calibration curves.

For the CBS-MS and sequential LC-MS, 0.75 mL urine sample was diluted with 0.75 mL of PBS (pH 7.4) in 2 mL of the 96 well-plate and agitated for 5 min at 1500 rpm. 96 blades were inserted into the blade holder and put on the automated system. The SPME process mainly consisted of the following three steps: 1) preconditioning, which involved placing the blades in an MeOH/water (50/50, v/v) solution and agitating them for 20 min at 1500 rpm; 2) extraction, which entailed immersing the blades in the sample solutions for 20 min with agitation at 1500 rpm; and 3) washing the blades with water for 10 s following the extraction step to eliminate the nonspecific attachment of salts or other impurities from the sample matrix. All the above three steps were done automatically with 96 samples simultaneously. After these, the blade was inserted into the CBS interface. 5 μL of the desorption solution (MeOH/ACN/H_2_O 85/10/5, v/v/v with 0.1% FA) was added on the top of the blade, desorbing for 12 s, and then 5 kV of the voltage was applied on the blade, generate a stable ESI spray for 9 s and detected by MS. After CBS-MS, sequential analysis was done by LC-MS. The blades were desorbed again in 200 µL of MeOH/ACN/H_2_O 85/10/5, v/v/v with 0.1% FA for 20 min. The desorption solution was dried using centrifuge concentrator for 30 min, and then reconstituted with 100 µL of MeOH/H_2_O 5/95, v/v with 0.1% FA solution, and then 10 µL of this solution was injected for LC-MS analysis. For making the matrix-match calibration curves, 5 urine samples from different volunteers were mixed as matrix blank and then spiked with 0%, 0.2%, 1%, 2%, 5%, 10%, 20%, 50%, 100%, 150% of MRPL standard mixture. There were 4 replicates at 0%, 100% and 150% concentration levels, and 7 replicates at other concentration levels. The IS concentration was at 1/4 MRPL and was spiked into the PBS solution in advance before sample dilution. The samples were operated according to general protocol for CBS-MS first and then LC-MS. SPME-LC-MS without CBS-MS in the first step was done for method comparison, 4 replicates were done for each calibration level.

For method validation, 12 urine samples from different volunteers were analyzed. For the reliability, the above 12 urine samples were spiked with the drugs at MRPL and then performed the analysis. For the testing of the accuracy (recovery), the urine was spiked at 25%, 50% and 100% MPRL with 7 replicates and then performed the analysis. For the testing of precision (reproducibility), the urine samples were spiked at 50% MRPL with 7 replicates (Intra-day RSD%) and performed the analysis in continuously three days (inter-day RSD%).

**Sample collection and research ethics.** All the protocols related to human urine samples were approved by University of Waterloo Research Ethics Boards, the project number is 30141. The Research Ethics was further approved by World Anti-doping Agency (WADA) before the project (22A10JP) started. For collection of human urine samples, informed written consent from all participants or next of kin was obtained prior to the research.

***
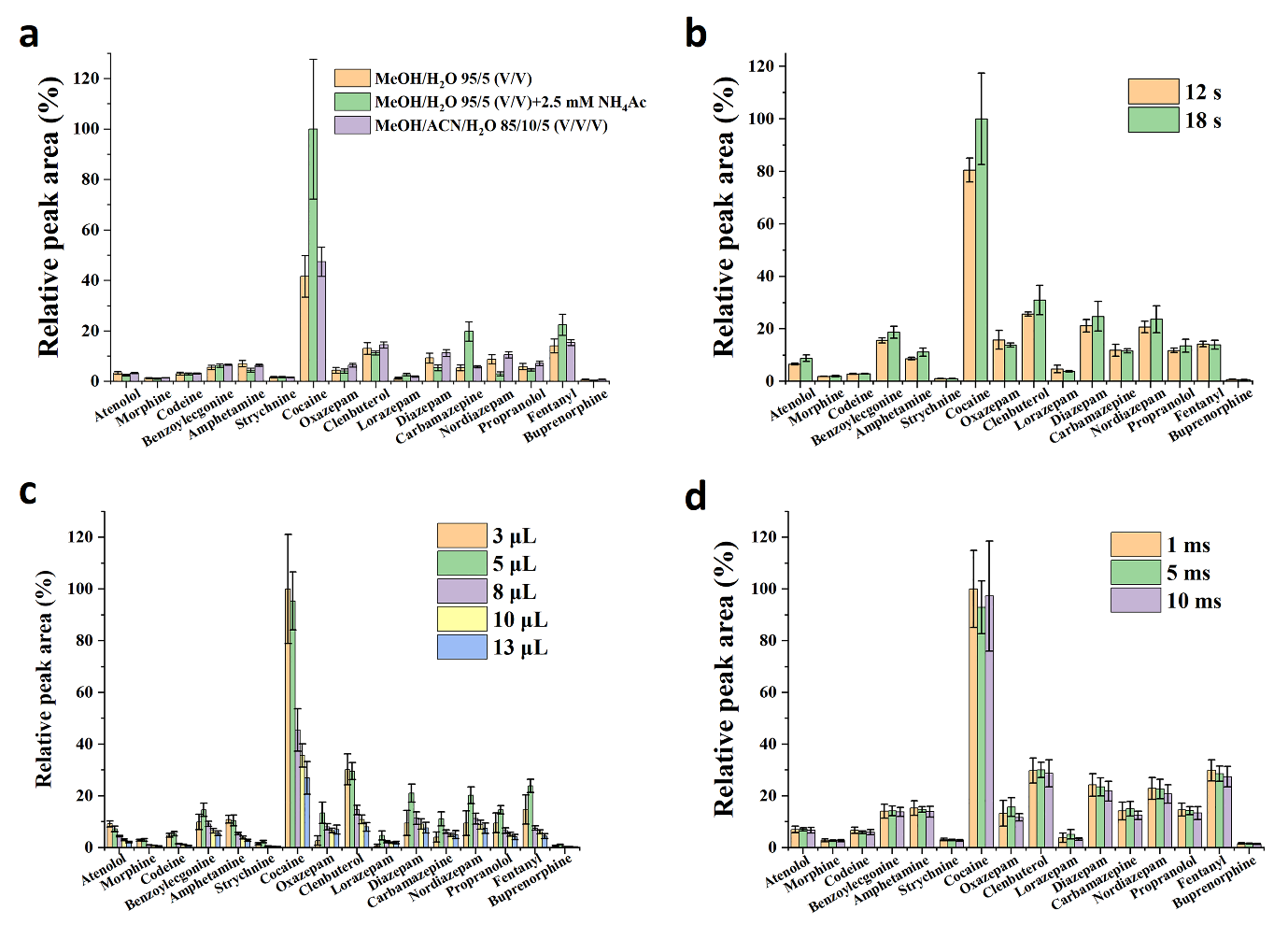
Figure
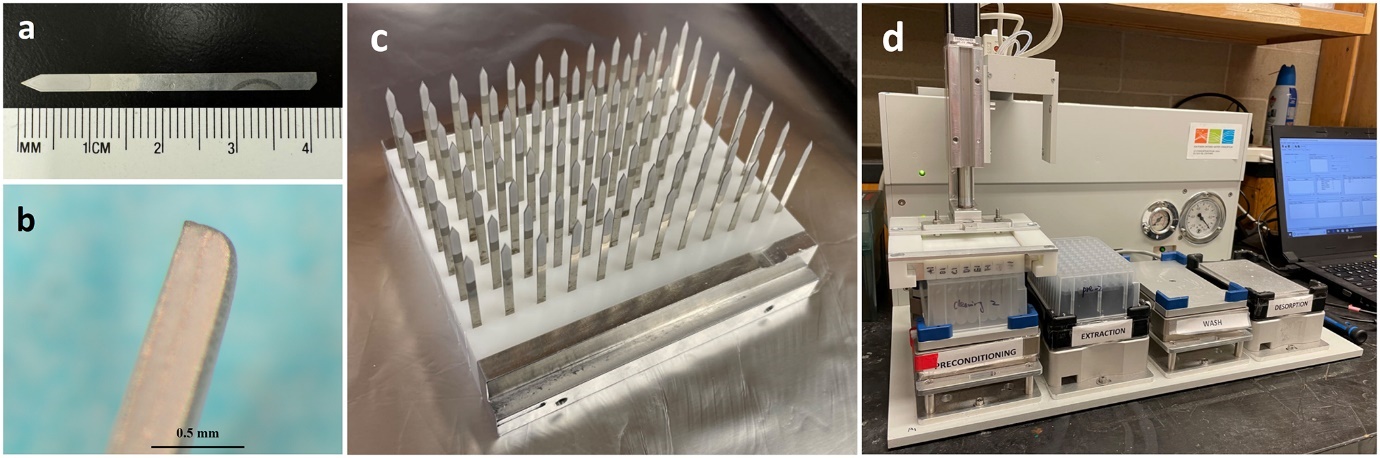
 S1.*** (a) The photo of CBS blade with HLB/PAN coating. (b) The microscope photo of the spray tip of the CBS blade. (c) 96 CBS blades on inserted into the holder. (d) The high throughput and automated SPME system.

***Figure S2.*** The optimization of desorption solution (a), desorption time (b), volume of the desorption solution (c) and the dwell time (d) for CBS-MS method. Data points and error bars represent mean values ± standard deviation (SD) of four technical replicates (n=4).


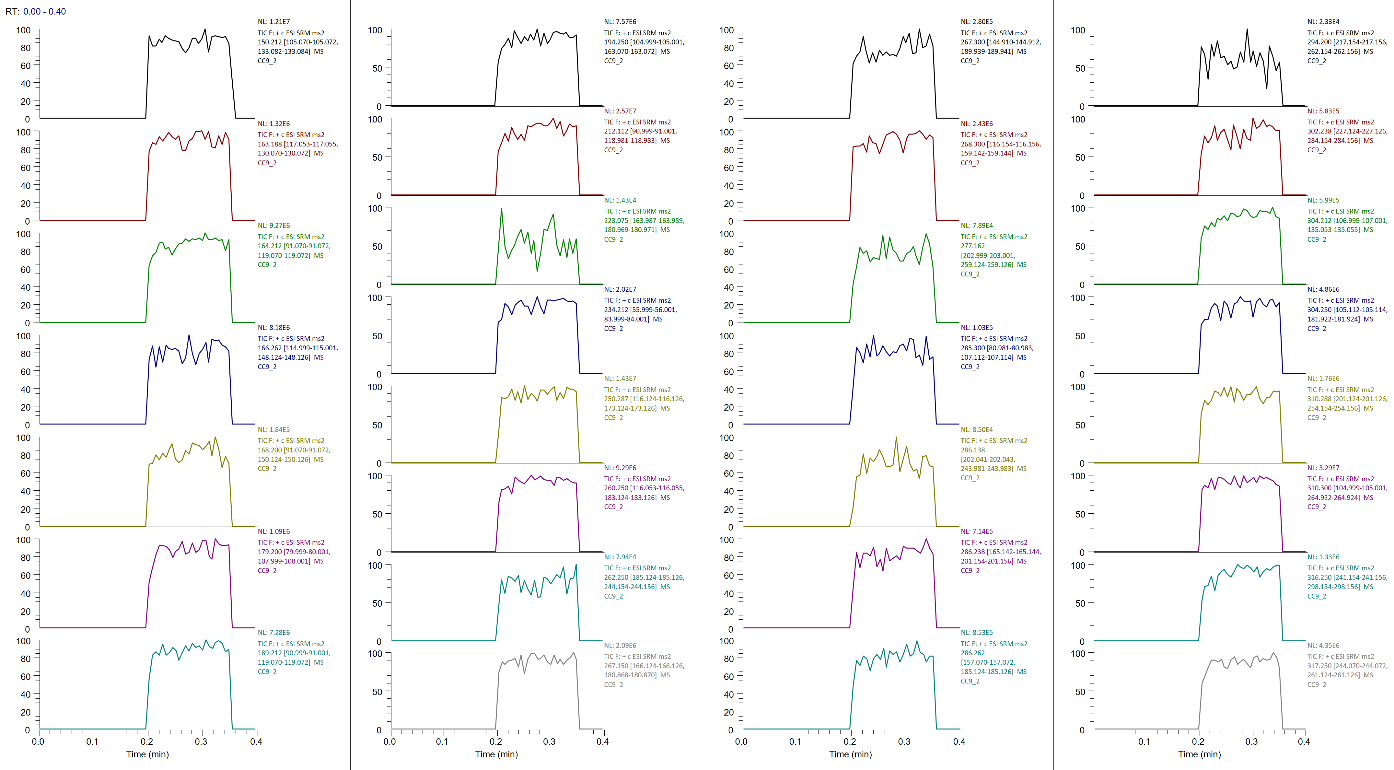

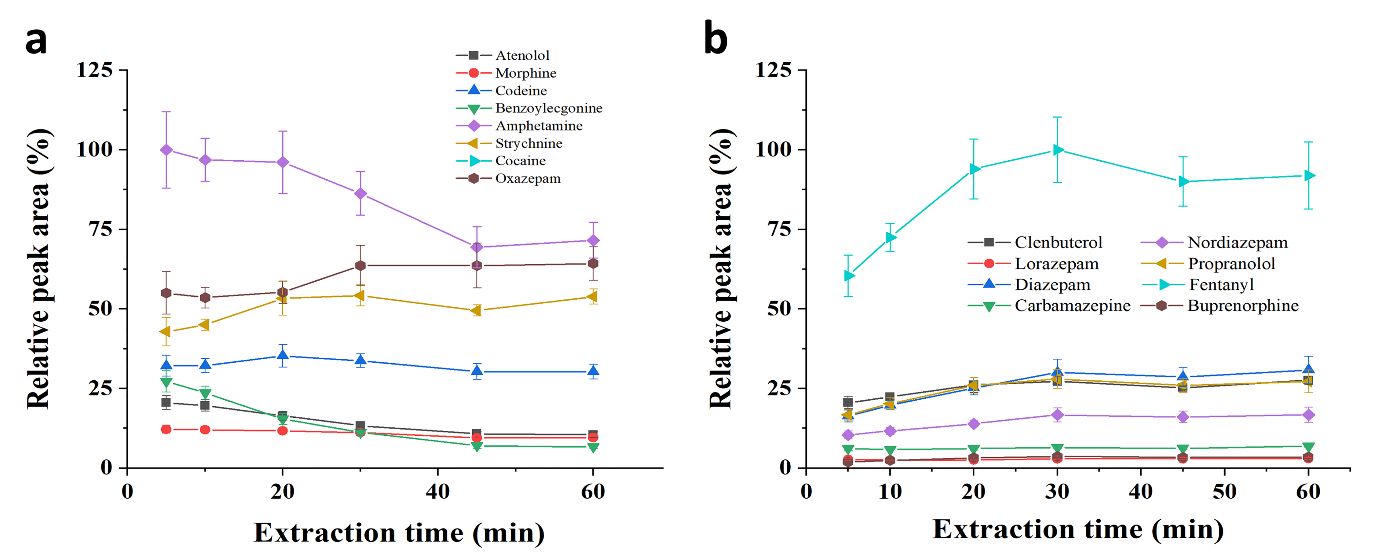
***Figure S3.*** The extraction time profile. Analytes were separated to two groups based on the relative peak area. Data points and error bars represent mean values ± standard deviation (SD) of four technical replicates (n=4).

***Figure S4.*** The representative CBS-MS spectrum for the targeted prohibited substances (Part A).

***
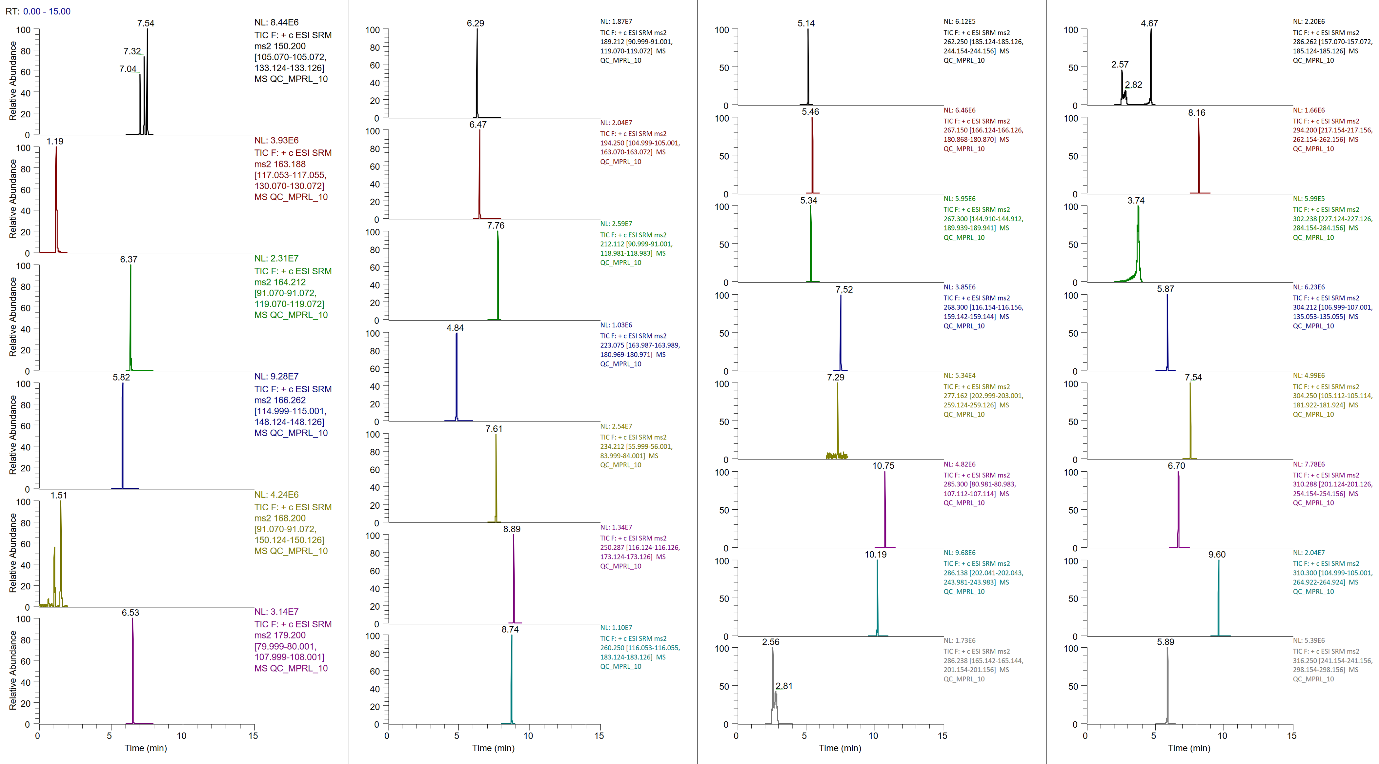

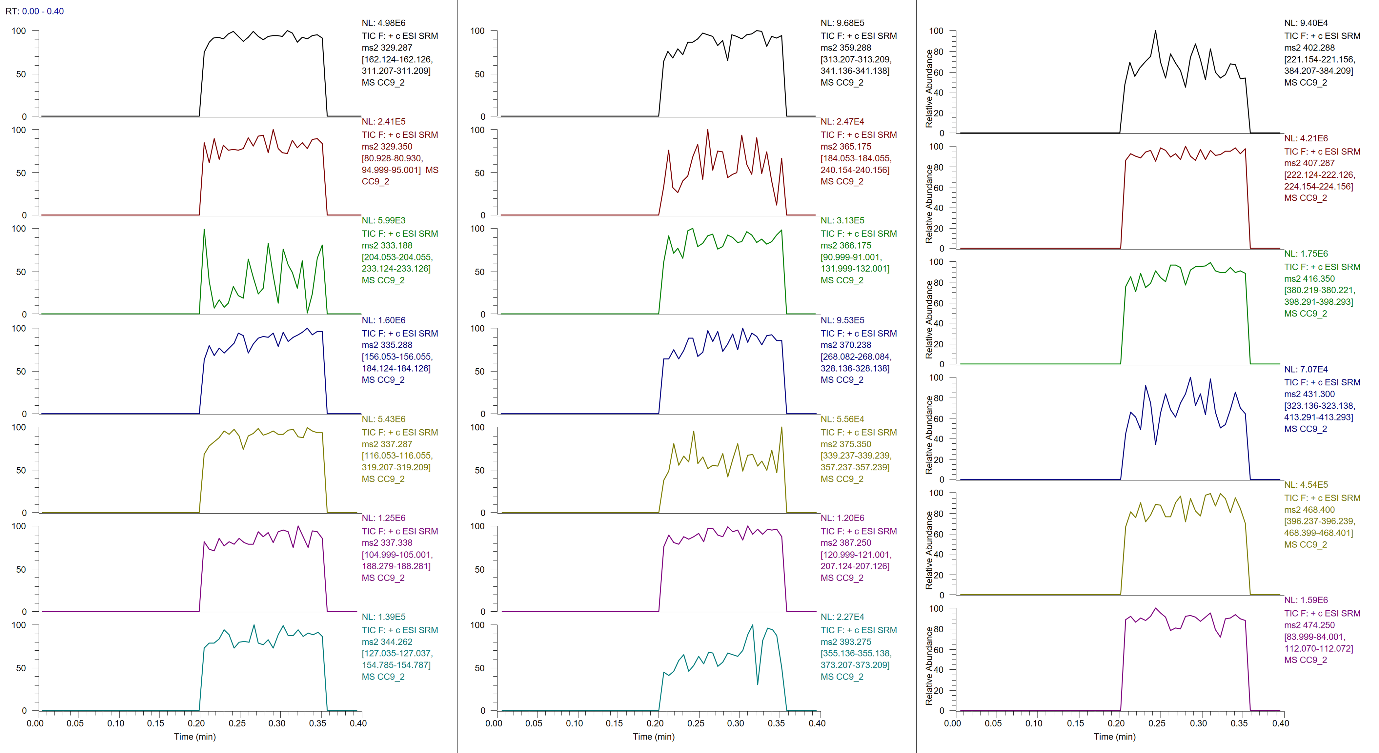
Figure S5.*** The representative CBS-MS spectrum for the targeted prohibited substances (Part B).

***Figure S6.*** The representative LC-MS chromatograms for the targeted prohibited substances (Part A).

***
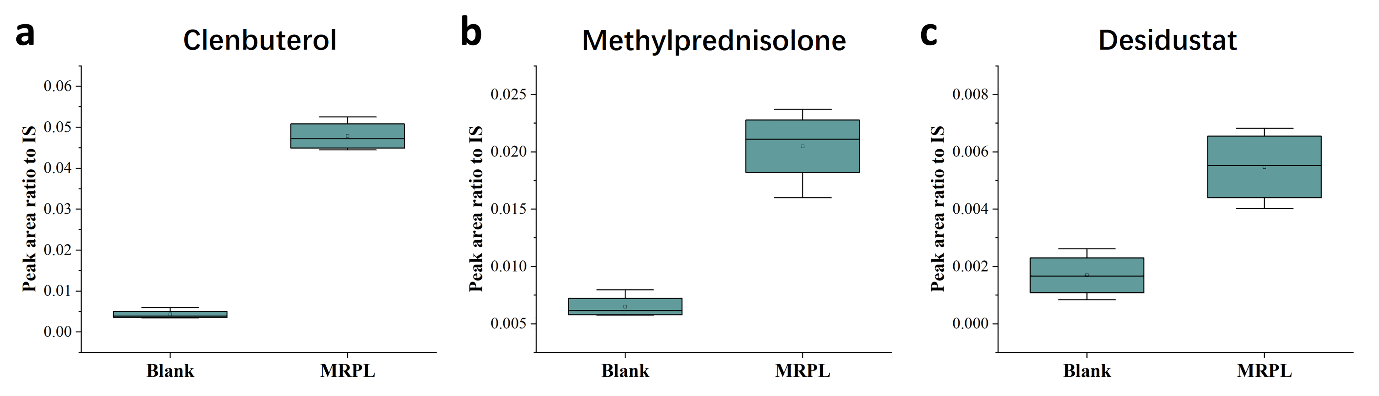
*
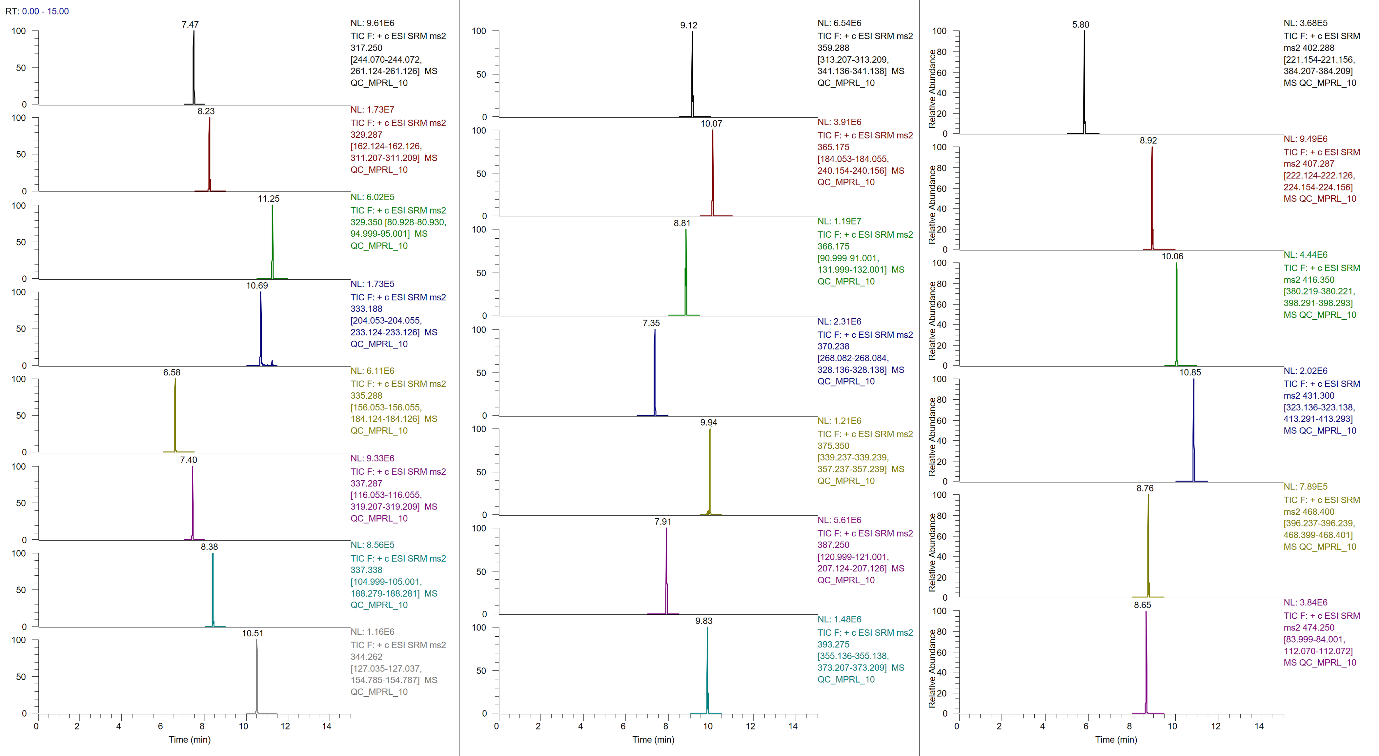
*Figure S7.*** The representative LC-MS chromatograms for the targeted prohibited substances (Part B).

***
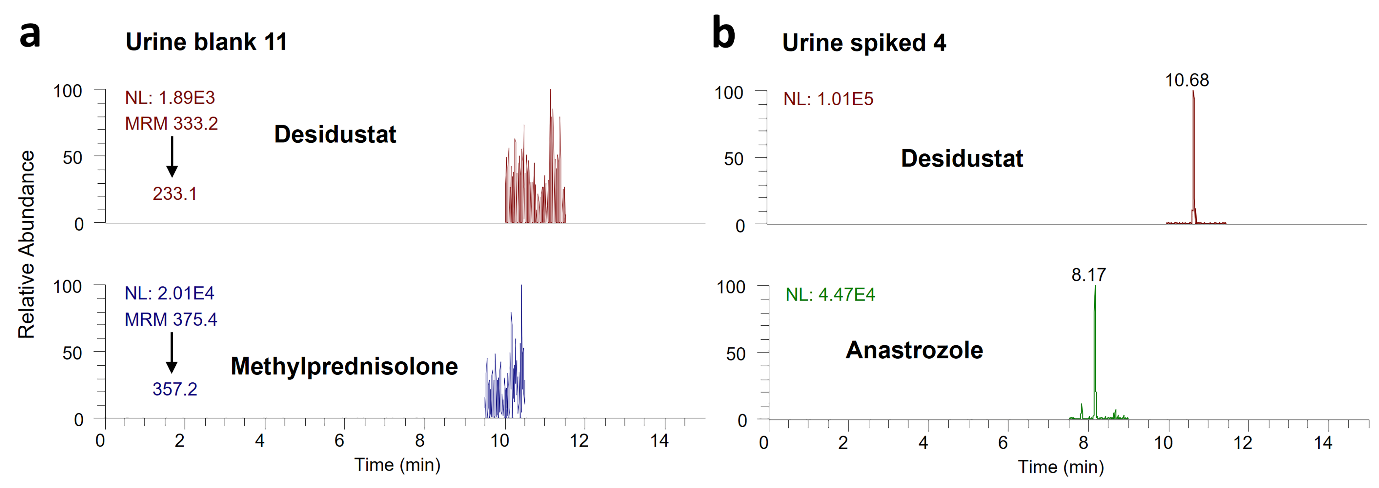
Figure S8.*** The boxing figures of clenbuterol (a), methylprednisolone (b) and desidustat (c) from urine blank and spiked urine samples at MRPL. Data points and error bars represent mean values ± standard deviation (SD) of four technical replicates (n=4).

***Figure S9.*** (a) The chromatograms of desidustat and methylprednisolone in blank urine 11 from the sequential LC-MS confirmation. (b) The chromatograms of desidustat and anastrozole in spiked urine 4 from the sequential LC-MS confirmation.

**Table S1.** Compare the amount of analytes in LC-MS desorption solution with or without CBS-MS

| Log P | Compounds | Without CBS | | After CBS | | Ratio |
| --- | --- | --- | --- | --- | --- | --- |
|  |  | Amount (ng) | RSD% (n=4) | Amount (ng) | RSD% (n=4) |  |
| 0.2 | Atenolol | 1.8 | 12% | 1.1 | 9% | 62% |
| 0.9 | Morphine | 1.1 | 8% | 0.7 | 16% | 64% |
| 1.2 | Codeine | 1.7 | 4% | 1.3 | 12% | 75% |
| 1.3 | Benzoylecgonine | 2.6 | 6% | 1.9 | 9% | 72% |
| 1.8 | Amphetamine | 1.4 | 16% | 1.0 | 6% | 74% |
| 1.9 | Strychnine | 1.3 | 12% | 0.9 | 11% | 69% |
| 2 | Cocaine | 3.8 | 1% | 3.5 | 4% | 93% |
| 2.2 | Oxazepam | 5.0 | 2% | 4.2 | 11% | 84% |
| 2.3 | Clenbuterol | 5.0 | 3% | 3.9 | 9% | 77% |
| 2.4 | Lorazepam | 5.6 | 2% | 4.4 | 9% | 79% |
| 2.6 | Diazepam | 5.8 | 2% | 5.1 | 8% | 88% |
| 2.8 | Carbamazepine | 4.7 | 1% | 3.9 | 10% | 83% |
| 2.9 | Nordiazepam | 5.2 | 1% | 4.4 | 8% | 84% |
| 3 | Propranolol | 4.8 | 5% | 4.2 | 7% | 87% |
| 4.1 | Fentanyl | 2.6 | 6% | 2.0 | 8% | 76% |
| 4.5 | Buprenorphine | 1.8 | 5% | 1.4 | 10% | 76% |

**Table S2.** Log P values and MS/MS parameters of the prohibited substances and internal standards.

| Compound | Log P | Precursor (m/z) | Product (m/z) | Collision Energy (V) | RF Lens (V) |
| --- | --- | --- | --- | --- | --- |
| P1-Acebutolol | 1.7 | 337.3 | 116.1 | 21.68 | 70 |
|  |  | 337.3 | 319.2 | 15.62 | 70 |
| P1-Alprenolol | 3.1 | 250.3 | 116.1 | 16.96 | 55 |
|  |  | 250.3 | 173.1 | 16.71 | 55 |
| P1-Atenolol | 0.2 | 267.3 | 189.9 | 15.66 | 61 |
|  |  | 267.3 | 144.9 | 29.26 | 61 |
| P1-Atenolol-d7 |  | 274.0 | 145.1 | 27.41 | 56 |
| P1-Carvedilol | 4.2 | 407.3 | 224.2 | 21.72 | 78 |
|  |  | 407.3 | 222.1 | 24.55 | 78 |
| P1-Labetalol | 2.7 | 329.3 | 311.2 | 12.67 | 53 |
|  |  | 329.3 | 162.1 | 25.26 | 53 |
| P1-Metoprolol | 1.9 | 268.3 | 159.1 | 20.92 | 57 |
|  |  | 268.3 | 116.2 | 18.31 | 57 |
| P1-Metoprolol-d7 |  | 275.3 | 123.2 | 19.28 | 62 |
| P1-Nadolol | 0.7 | 310.3 | 254.2 | 16.71 | 55 |
|  |  | 310.3 | 201.1 | 22.40 | 55 |
| P1-Propranolol | 3.5 | 260.3 | 116.1 | 17.93 | 56 |
|  |  | 260.3 | 183.1 | 17.85 | 56 |
| P1-Propranolol-d7 |  | 267.3 | 189.2 | 18.35 | 59 |
| P1-Timolol | 1.8 | 317.3 | 261.1 | 15.95 | 53 |
|  |  | 317.3 | 244.1 | 21.43 | 53 |
| S1-Clenbuterol | 2.2 | 277.2 | 203.0 | 15.49 | 42 |
|  |  | 277.2 | 259.1 | 10.69 | 42 |
| S1-Clenbuterol-d9 |  | 286.2 | 204.0 | 16.71 | 45 |
| S1-Stanozolol | 4.4 | 329.4 | 95.0 | 41.39 | 103 |
|  |  | 329.4 | 80.9 | 44.39 | 103 |
| S1-Stanozolol-d3 |  | 332.4 | 81.0 | 45.23 | 105 |
| S1-Zilpaterol | 0.5 | 262.3 | 185.1 | 22.78 | 46 |
|  |  | 262.3 | 244.2 | 12.29 | 46 |
| S2-Desidustat | 1.9 | 333.2 | 233.1 | 14.60 | 52 |
|  |  | 333.2 | 204.1 | 25.01 | 52 |
| S3-Fenoterol | 2.0 | 304.2 | 135.1 | 18.02 | 57 |
|  |  | 304.2 | 107.0 | 29.98 | 57 |
| S3-Fenoterol-d6 |  | 310.3 | 141.1 | 18.56 | 57 |
| S3-Olodaterol | 1.8 | 387.3 | 207.1 | 19.24 | 61 |
|  |  | 387.3 | 121.0 | 34.06 | 61 |
| S3-Olodaterol-d3 |  | 390.3 | 207.1 | 19.32 | 66 |
| S3-Reproterol | -0.5 | 402.3 | 221.2 | 26.74 | 65 |
|  |  | 402.3 | 384.2 | 16.20 | 65 |
| S3-Salmeterol | 4.2 | 416.4 | 398.3 | 14.48 | 62 |
|  |  | 416.4 | 380.2 | 18.61 | 62 |
| S4-Anastrozole | 2.1 | 294.2 | 217.2 | 21.01 | 72 |
|  |  | 294.2 | 262.2 | 11.23 | 72 |
| S4-Arimistane | 3.2 | 285.3 | 81.0 | 27.07 | 67 |
|  |  | 285.3 | 107.1 | 27.20 | 67 |
| S4-Raloxifene | 6.1 | 474.3 | 112.1 | 29.89 | 110 |
|  |  | 474.3 | 84.0 | 41.39 | 110 |
| S4-Trimetazidine | 1.0 | 267.2 | 180.9 | 14.39 | 48 |
|  |  | 267.2 | 166.1 | 24.88 | 48 |
| S4-Trimetazidine-d8 |  | 275.3 | 181.1 | 15.03 | 38 |
| S5-Acetazolamide | -0.3 | 223.1 | 181.0 | 14.77 | 53 |
|  |  | 223.1 | 164.0 | 22.02 | 53 |
| S5-Bumetanide | 2.8 | 365.2 | 184.1 | 22.27 | 68 |
|  |  | 365.2 | 240.2 | 16.92 | 68 |
| S5-Bumetanide-d5 |  | 370.2 | 244.1 | 17.22 | 73 |
| S5-Indapamide | 2.2 | 366.2 | 132.0 | 14.69 | 59 |
|  |  | 366.2 | 91.0 | 38.15 | 59 |
| S5-Probenecid | 3.2 | 286.1 | 202.0 | 15.53 | 50 |
|  |  | 286.1 | 244.0 | 12.25 | 50 |
| S5-Pseudoephedrine | 0.9 | 166.3 | 115.0 | 28.00 | 30 |
|  |  | 166.3 | 148.1 | 12.25 | 30 |
| S6-Cocaine | 2.3 | 304.3 | 181.9 | 19.24 | 59 |
|  |  | 304.3 | 105.1 | 32.25 | 59 |
| S6-Cocaine-d3 |  | 307.3 | 185.1 | 19.49 | 62 |
| S6-Dimethylamphetamine | 2.7 | 164.2 | 91.1 | 21.51 | 36 |
|  |  | 164.2 | 119.1 | 12.79 | 36 |
| S6-Fenproporex | 1.9 | 189.2 | 91.0 | 20.59 | 30 |
|  |  | 189.2 | 119.1 | 10.27 | 30 |
| S6-MDMA | 2.2 | 194.3 | 163.1 | 12.29 | 31 |
|  |  | 194.3 | 105.0 | 24.00 | 31 |
| S6-MDMA-d5 |  | 199.0 | 165.1 | 12.96 | 37 |
| S6-Mefenorex | 3.1 | 212.1 | 91.0 | 22.99 | 41 |
|  |  | 212.1 | 119.0 | 13.38 | 41 |
| S6-Phentermine | 1.9 | 150.2 | 105.1 | 17.51 | 30 |
|  |  | 150.2 | 133.1 | 8.92 | 30 |
| S6-Ortetamine | 2.1 | 150.2 | 105.1 | 17.51 | 30 |
|  |  | 150.2 | 133.1 | 8.92 | 30 |
| S6-p-Methylamphetamine | 2.5 | 150.2 | 105.1 | 17.51 | 30 |
|  |  | 150.2 | 133.1 | 8.92 | 30 |
| S6-Methylphenidate | 0.2 | 234.2 | 84.0 | 20.46 | 47 |
|  |  | 234.2 | 56.0 | 40.85 | 47 |
| S6-Nicotine | 1.2 | 163.2 | 130.1 | 20.92 | 45 |
|  |  | 163.2 | 117.1 | 26.31 | 45 |
| S6-Nicotine-d4 |  | 167.2 | 121.1 | 27.54 | 33 |
| S6-Nikethamide | 0.3 | 179.2 | 108.0 | 18.82 | 51 |
|  |  | 179.2 | 80.0 | 28.50 | 51 |
| S6-Strychnine | 1.9 | 335.3 | 184.1 | 38.61 | 93 |
|  |  | 335.3 | 156.1 | 46.83 | 93 |
| S6-Synephrine | -0.6 | 168.2 | 150.1 | 9.25 | 30 |
|  |  | 168.2 | 91.1 | 22.27 | 30 |
| S6-Phenylephrine | -0.3 | 168.2 | 150.1 | 9.25 | 30 |
|  |  | 168.2 | 91.1 | 22.27 | 30 |
| S7-Buprenorphine | 5.0 | 468.4 | 468.4 | 35.16 | 105 |
|  |  | 468.4 | 396.2 | 39.58 | 105 |
| S7-Buprenorphine-d4 |  | 472.4 | 472.4 | 40.38 | 110 |
| S7-Fentanyl | 4.1 | 337.3 | 188.3 | 22.78 | 68 |
|  |  | 337.3 | 105.0 | 37.14 | 68 |
| S7-Fentanyl-d5 |  | 342.3 | 188.1 | 23.16 | 69 |
| S7-Heroin | 1.6 | 370.2 | 328.1 | 26.06 | 84 |
|  |  | 370.2 | 268.1 | 28.21 | 84 |
| S7-Heroin-d9 |  | 379.3 | 272.2 | 29.30 | 85 |
| S7-Hydromorphone | 1.1 | 286.3 | 157.1 | 42.15 | 74 |
|  |  | 286.3 | 185.1 | 30.48 | 74 |
| S7-Hydromorphone-d3 |  | 289.3 | 185.1 | 30.53 | 65 |
| S7-Methadone | 3.9 | 310.3 | 264.9 | 14.22 | 49 |
|  |  | 310.3 | 105.0 | 27.54 | 49 |
| S7-Methadone-d3 |  | 313.3 | 268.2 | 14.77 | 48 |
| S7-Morphine | 0.9 | 286.2 | 165.1 | 39.84 | 75 |
|  |  | 286.2 | 201.2 | 25.51 | 75 |
| S7-Morphine-d3 |  | 289.3 | 165.1 | 41.44 | 74 |
| S7-Oxycodone | 0.7 | 316.3 | 241.2 | 28.80 | 57 |
|  |  | 316.3 | 298.2 | 18.56 | 57 |
| S7-Oxycodone-d3 |  | 319.3 | 244.2 | 28.63 | 58 |
| S7-Oxymorphone | 0.8 | 302.2 | 227.1 | 29.81 | 58 |
|  |  | 302.2 | 284.2 | 19.45 | 58 |
| S8-JWH-073 | 5.8 | 344.3 | 127.0 | 55.00 | 70 |
|  |  | 344.3 | 154.8 | 20.84 | 70 |
| S9-Budesonide | 1.9 | 431.3 | 323.1 | 12.75 | 51 |
|  |  | 431.3 | 413.3 | 10.81 | 51 |
| S9-Dexamethasone | 1.8 | 393.3 | 355.1 | 11.11 | 39 |
|  |  | 393.3 | 373.2 | 8.67 | 39 |
| S9-Methylprednisolone | 1.8 | 375.4 | 357.2 | 10.35 | 40 |
|  |  | 375.4 | 339.2 | 10.27 | 40 |
| S9-Prednisone | 1.5 | 359.3 | 313.2 | 12.12 | 51 |
|  |  | 359.3 | 341.1 | 10.94 | 51 |

**Table S3.** The linearity data about CBS-MS.

| Compound | MRPL^1^ | CBS-MS | | | | |
| --- | --- | --- | --- | --- | --- | --- |
|  |  | Slope | Intercept | R^2^ | LOQ^1^ | Linearity range^1^ |
| P1-Acebutolol | 50.0 | 6.7119 | -0.0497 | 0.9996 | 0.1 | 0.1-75 |
| P1-Alprenolol | 50.0 | 38.9813 | -0.0012 | 0.9996 | 0.1 | 0.1-75 |
| P1-Atenolol | 50.0 | 2.2069 | 0.0175 | 0.9996 | 25 | 25-75 |
| P1-Carvedilol | 50.0 | 8.5497 | -0.0949 | 0.9991 | 0.1 | 0.1-75 |
| P1-Labetalol | 50.0 | 11.2514 | 0.1461 | 0.9996 | 2.5 | 2.5-75 |
| P1-Metoprolol | 50.0 | 1.4747 | -0.0146 | 0.9995 | 5 | 5-75 |
| P1-Nadolol | 50.0 | 0.9581 | -0.0058 | 0.9997 | 0.5 | 0.5-75 |
| P1-Propranolol | 50.0 | 17.3404 | -0.0699 | 1.0000 | 0.1 | 0.1-75 |
| P1-Timolol | 50.0 | 4.9549 | 0.0205 | 0.9993 | 0.5 | 0.5-75 |
| S1-Clenbuterol | 0.2 | / | / | / | 0.3 | / |
| S1-Stanozolol | 2.5 | 0.1964 | 0.0019 | 0.9953 | 1.25 | 1.25-3.75 |
| S1-Zilpaterol | 2.0 | 0.9981 | -0.0327 | 0.9977 | 1 | 1-100 |
| S2-Desidustat | 2.0 | / | / | / | / | / |
| S3-Fenoterol | 20.0 | 0.4706 | -0.0022 | 0.9990 | 1 | 1-30 |
| S3-Olodaterol | 20.0 | 4.3868 | -0.0203 | 0.9999 | 0.4 | 0.4-30 |
| S3-Reproterol | 20.0 | 0.5283 | -0.0290 | 0.9983 | 10 | 10-30 |
| S3-Salmeterol | 10.0 | 0.0240 | 0.0003 | 0.9975 | 0.2 | 0.2-15 |
| S4-Anastrozole | 20.0 | / | / | / | / | / |
| S4-Arimistane | 20.0 | / | / | / | 30 | / |
| S4-Raloxifene | 20.0 | 3.7691 | -0.0363 | 0.9996 | 0.2 | 0.2-30 |
| S4-Trimetazidine | 20.0 | 4.3751 | -0.1095 | 0.9991 | 0.4 | 0.4-30 |
| S5-Acetazolamide | 20.0 | / | / | / | / | / |
| S5-Bumetanide | 20.0 | 0.0002 | 0.0000 | 0.9971 | 10 | 10-30 |
| S5-Indapamide | 200.0 | 0.7018 | -0.0017 | 0.9995 | 2 | 2-300 |
| S5-Probenecid | 200.0 | 0.1068 | 0.0020 | 0.9979 | 2 | 2-300 |
| S5-Pseudoephedrine | 200.0 | 4.2035 | 0.0296 | 0.9993 | 0.4 | 0.4-300 |
| S6-Cocaine | 10.0 | 20.3590 | 0.0682 | 0.9967 | 0.02 | 0.02-15 |
| S6-Dimethylamphetamine | 50.0 | 5.5082 | -0.0471 | 0.9997 | 0.5 | 0.5-75 |
| S6-Fenproporex | 20.0 | 3.8088 | -0.0280 | 0.9999 | 0.2 | 0.2-30 |
| S6-MDMA | 20.0 | 4.0452 | -0.0160 | 0.9999 | 0.04 | 0.04-30 |
| S6-Mefenorex | 20.0 | 137.8505 | 0.5498 | 0.9986 | 0.04 | 0.04-30 |
| S6-Phentermine | 50.0 | 14.6951 | 0.0588 | 0.9998 | 0.3 | 0.3-225 |
| S6-Ortetamine | 50.0 |  |  |  |  |  |
| S6-p-Methylamphetamine | 50.0 |  |  |  |  |  |
| S6-Methylphenidate | 50.0 | 121.7409 | 0.5628 | 0.9993 | 0.1 | 0.1-75 |
| S6-Nicotine | 50.0 | 7.8510 | -0.1252 | 0.9978 | 0.5 | 0.5-75 |
| S6-Nikethamide | 50.0 | 0.6628 | -0.0117 | 0.9997 | 1 | 1-75 |
| S6-Strychnine | 50.0 | 0.6702 | -0.0070 | 0.9994 | 0.5 | 0.5-75 |
| S6-Synephrine | 50.0 | / | / | / | / | / |
| S6-Phenylephrine | 50.0 |  |  |  |  |  |
| S7-Buprenorphine | 2.5 | 0.0952 | -0.0011 | 0.9993 | 0.05 | 0.05-3.75 |
| S7-Fentanyl | 1.0 | 2.2734 | -0.0117 | 0.9998 | 0.01 | 0.01-1.5 |
| S7-Heroin | 25.0 | 4.7974 | -0.0512 | 0.9996 | 0.25 | 0.25-37.5 |
| S7-Hydromorphone | 25.0 | 5.6794 | 0.0054 | 0.9993 | 0.5 | 0.5-37.5 |
| S7-Methadone | 25.0 | 0.8382 | -0.0009 | 1.0000 | 0.05 | 0.05-37.5 |
| S7-Morphine^2^ | 1000.0 | 8.8065 | -0.0863 | 0.9999 | 4 | 4-300 |
| S7-Oxycodone | 25.0 | 5.7361 | 0.0530 | 0.9985 | 1.25 | 1.25-37.5 |
| S7-Oxymorphone | 25.0 | 1.3368 | 0.0239 | 0.9995 | 2.5 | 2.5-37.5 |
| S8-JWH-073 | 1.0 | 0.2012 | -0.0355 | 0.9948 | 0.5 | 0.5-1.5 |
| S9-Budesonide | 45.0 | 0.0226 | 0.0010 | 0.9934 | 9 | 9-67.5 |
| S9-Dexamethasone | 60.0 | 0.0002 | 0.0000 | 0.9981 | 12 | 12-90 |
| S9-Methylprednisolone | 30.0 | / | / | / | / | / |
| S9-Prednisone | 300.0 | 0.1055 | 0.0002 | 0.9981 | 150 | 150-450 |

^1^The unit is ng/mL; ^2^For morphine, the MRPL is 1000 ng/mL, but for making the calibration curves, 200 ng/mL was used. The related internal standards for quantitation are the same as Table S4.

**Table S4.** The linearity data about sequential LC-MS after CBS-MS.

| Compound | Internal standard | Sequential LC-MS | | | | |
| --- | --- | --- | --- | --- | --- | --- |
|  |  | Slope | Intercept | R^2^ | LOQ^1^ | Linearity range^1^ |
| P1-Acebutolol | Metoprolol-d7 | 6.9308 | -0.0354 | 0.9996 | 0.1 | 0.1-75 |
| P1-Alprenolol | Buprenorphine-d4 | 25.9074 | 0.0125 | 0.9977 | 0.1 | 0.1-75 |
| P1-Atenolol | Atenolol-d7 | 4.7996 | -0.0486 | 0.9995 | 1 | 1-75 |
| P1-Carvedilol | Buprenorphine-d4 | 17.6983 | 0.0541 | 0.9955 | 0.1 | 0.1-75 |
| P1-Labetalol | Buprenorphine-d4 | 36.1395 | 0.3846 | 0.9910 | 0.5 | 0.5-75 |
| P1-Metoprolol | Metoprolol-d7 | 1.5054 | -0.0054 | 0.9998 | 0.5 | 0.5-75 |
| P1-Nadolol | MDMA-d5 | 1.9590 | -0.0127 | 0.9961 | 0.5 | 0.5-75 |
| P1-Propranolol | Propranolol-d7 | 16.4369 | 0.0347 | 0.9996 | 0.1 | 0.1-75 |
| P1-Timolol | Metoprolol-d7 | 7.0029 | -0.0304 | 0.9994 | 0.1 | 0.1-75 |
| S1-Clenbuterol | Clenbuterol-d9 | 0.0422 | 0.0007 | 0.9999 | 0.04 | 0.04-0.3 |
| S1-Stanozolol | Stanozolol-d3 | 0.5459 | -0.0044 | 0.9998 | 0.025 | 0.025-3.75 |
| S1-Zilpaterol | Atenolol-d7 | 0.4593 | -0.0071 | 0.9980 | 0.1 | 0.1-100 |
| S2-Desidustat | Buprenorphine-d4 | 0.2013 | -0.0229 | 0.9955 | 1 | 1-3 |
| S3-Fenoterol | Fenoterol-d6 | 7.2678 | 0.0130 | 0.9996 | 0.2 | 0.2-30 |
| S3-Olodaterol | Olodaterol-d3 | 4.4716 | -0.0166 | 0.9992 | 0.2 | 0.2-30 |
| S3-Reproterol | Atenolol-d7 | 3.2065 | -0.0572 | 0.9951 | 0.4 | 0.4-30 |
| S3-Salmeterol | Methadone-d3 | 0.1599 | 0.0000 | 0.9985 | 0.1 | 0.1-15 |
| S4-Anastrozole | Olodaterol-d3 | 0.0362 | -0.0001 | 0.9999 | 10 | 10-30 |
| S4-Arimistane | Stanozolol-d3 | 5.3297 | -0.1267 | 0.9976 | 0.4 | 0.4-30 |
| S4-Raloxifene | Propranolol-d7 | 6.6536 | 0.1397 | 0.9974 | 0.4 | 0.4-30 |
| S4-Trimetazidine | Trimetazidine-d8 | 4.0373 | -0.0925 | 0.9995 | 0.2 | 0.2-30 |
| S5-Acetazolamide | / | / | / | / | / | / |
| S5-Bumetanide | Methadone-d3 | 0.0500 | 0.0002 | 0.9960 | 1 | 1-30 |
| S5-Indapamide | Buprenorphine-d4 | 21.7435 | 0.5173 | 0.9965 | 2 | 2-300 |
| S5-Probenecid | Propranolol-d7 | 4.8321 | 0.1015 | 0.9934 | 4 | 4-300 |
| S5-Pseudoephedrine | Trimetazidine-d8 | 28.7940 | 0.1733 | 0.9997 | 0.4 | 0.4-300 |
| S6-Cocaine | Cocaine-d3 | 22.3420 | -0.0612 | 0.9999 | 0.02 | 0.02-15 |
| S6-Dimethylamphetamine | MDMA-d5 | 3.7105 | -0.0158 | 0.9947 | 0.5 | 0.5-75 |
| S6-Fenproporex | MDMA-d5 | 6.6691 | -0.0240 | 0.9967 | 0.2 | 0.2-30 |
| S6-MDMA | MDMA-d5 | 4.4373 | 0.0065 | 0.9960 | 0.04 | 0.04-30 |
| S6-Mefenorex | Olodaterol-d3 | 22.3507 | 0.2344 | 0.9931 | 0.04 | 0.04-30 |
| S6-Phentermine | Clenbuterol-d9 | 0.0805 | 0.0057 | 0.9868 | 10 | 10-75 |
| S6-Ortetamine | Clenbuterol-d9 | 8.2222 | 0.1409 | 0.9948 | 0.5 | 0.5-75 |
| S6-p-Methylamphetamine | Clenbuterol-d9 | 10.2342 | 0.1839 | 0.9965 | 0.5 | 0.5-75 |
| S6-Methylphenidate | Olodaterol-d3 | 17.2775 | 0.2142 | 0.9916 | 0.1 | 0.1-75 |
| S6-Nicotine | Nicotine-d4 | 7.2020 | -0.1256 | 0.9993 | 2.5 | 2.5-75 |
| S6-Nikethamide | MDMA-d5 | 3.1431 | -0.0527 | 0.9983 | 0.5 | 0.5-75 |
| S6-Strychnine | MDMA-d5 | 1.5715 | -0.0059 | 0.9984 | 0.5 | 0.5-75 |
| S6-Synephrine | / | / | / | / | / | / |
| S6-Phenylephrine | / | / | / | / | / | / |
| S7-Buprenorphine | Buprenorphine-d4 | 1.6082 | -0.0126 | 0.9999 | 0.05 | 0.05-3.75 |
| S7-Fentanyl | Fentanyl-d5 | 2.1407 | -0.0026 | 0.9999 | 0.01 | 0.01-1.5 |
| S7-Heroin | Heroin-d9 | 6.9862 | -0.0758 | 0.9996 | 0.25 | 0.25-37.5 |
| S7-Hydromorphone | Hydromorphone-d3 | 5.6623 | -0.0216 | 0.9999 | 1.25 | 1.25-37.5 |
| S7-Methadone | Methadone-d3 | 0.9567 | 0.0000 | 0.9999 | 0.25 | 0.25-37.5 |
| S7-Morphine^2^ | Morphine-d3 | 8.6278 | -0.0998 | 0.9999 | 10 | 10-300 |
| S7-Oxycodone | Oxycodone-d3 | 26.9713 | -0.4270 | 0.9993 | 0.5 | 0.5-37.5 |
| S7-Oxymorphone | Hydromorphone-d3 | 4.5442 | -0.1989 | 0.9998 | 2.5 | 2.5-37.5 |
| S8-JWH-073 | Buprenorphine-d4 | 2.0086 | -0.0621 | 0.9978 | 0.1 | 0.1-1.5 |
| S9-Budesonide | Stanozolol-d3 | 0.6910 | -0.0221 | 0.9984 | 2.25 | 2.25-67.5 |
| S9-Dexamethasone | Methadone-d3 | 0.0409 | 0.0004 | 0.9965 | 3 | 3-90 |
| S9-Methylprednisolone | Buprenorphine-d4 | 0.0358 | 0.0029 | 0.9946 | 6 | 6-45 |
| S9-Prednisone | Buprenorphine-d4 | 4.2042 | 0.4939 | 0.9894 | 30 | 30-450 |

^1^The unit is ng/mL; ^2^For morphine, the MRPL is 1000 ng/mL, but for making the calibration curves, 200 ng/mL was used.

**Table S5.** Linear information about standalone SPME-LC-MS.

| Compound | SPME-LC-MS | | | | |
| --- | --- | --- | --- | --- | --- |
|  | Slope | Intercept | R^2^ | LOQ^1^ | Linearity range^1^ |
| P1-Acebutolol | 6.1246 | 0.1447 | 0.9947 | 0.1 | 0.1-75 |
| P1-Alprenolol | 21.9015 | 0.0131 | 0.9966 | 0.1 | 0.1-75 |
| P1-Atenolol | 5.0804 | -0.1163 | 0.9991 | 1 | 1-75 |
| P1-Carvedilol | 16.3086 | -0.0874 | 0.9998 | 0.1 | 0.1-75 |
| P1-Labetalol | 31.4604 | 0.0474 | 0.9999 | 0.5 | 0.5-75 |
| P1-Metoprolol | 1.5243 | -0.0015 | 1.0000 | 0.5 | 0.5-75 |
| P1-Nadolol | 1.8077 | -0.0215 | 0.9994 | 0.5 | 0.5-75 |
| P1-Propranolol | 16.3527 | 0.1633 | 0.9992 | 0.1 | 0.1-75 |
| P1-Timolol | 6.9959 | -0.0316 | 0.9967 | 0.1 | 0.1-75 |
| S1-Clenbuterol | 0.0428 | 0.0005 | 0.9918 | 0.04 | 0.04-0.3 |
| S1-Stanozolol | 0.5746 | -0.0088 | 0.9994 | 0.025 | 0.025-3.75 |
| S1-Zilpaterol | 0.4147 | -0.0041 | 0.9976 | 0.1 | 0.1-100 |
| S2-Desidustat | 0.0800 | -0.0077 | 0.9998 | 1 | 1-3 |
| S3-Fenoterol | 4.7830 | -0.0160 | 1.0000 | 0.2 | 0.2-30 |
| S3-Olodaterol | 4.5925 | -0.0315 | 0.9999 | 0.2 | 0.2-30 |
| S3-Reproterol | 2.3148 | -0.0531 | 0.9945 | 0.4 | 0.4-30 |
| S3-Salmeterol | 0.1770 | -0.0048 | 0.9991 | 0.1 | 0.1-15 |
| S4-Anastrozole | 0.0402 | -0.0099 | 0.9851 | 10 | 10-30 |
| S4-Arimistane | 2.7586 | 0.0012 | 0.9994 | 0.4 | 0.4-30 |
| S4-Raloxifene | 8.6926 | -0.0565 | 0.9993 | 0.4 | 0.4-30 |
| S4-Trimetazidine | 4.1049 | -0.1019 | 0.9986 | 0.2 | 0.2-30 |
| S5-Acetazolamide | / | / | / | / | / |
| S5-Bumetanide | 0.0300 | -0.0013 | 0.9993 | 1 | 1-30 |
| S5-Indapamide | 16.7816 | 0.1605 | 0.9995 | 2 | 2-300 |
| S5-Probenecid | 4.1578 | -0.0044 | 0.9949 | 4 | 4-300 |
| S5-Pseudoephedrine | 6.3801 | -0.0220 | 0.9966 | 0.4 | 0.4-300 |
| S6-Cocaine | 21.8328 | 0.1249 | 0.9995 | 0.02 | 0.02-15 |
| S6-Dimethylamphetamine | 4.6640 | -0.0584 | 0.9989 | 0.5 | 0.5-75 |
| S6-Fenproporex | 7.1320 | -0.0620 | 0.9996 | 0.04 | 0.04-30 |
| S6-MDMA | 4.5029 | -0.0209 | 0.9999 | 0.04 | 0.04-30 |
| S6-Mefenorex | 22.6466 | -0.0630 | 0.9977 | 0.04 | 0.04-30 |
| S6-Phentermine | 0.0893 | -0.0012 | 0.9976 | 5 | 5-75 |
| S6-Ortetamine | 8.7596 | 0.0360 | 0.9981 | 0.1 | 0.1-75 |
| S6-p-Methylamphetamine | 10.2674 | 0.2476 | 0.9964 | 0.5 | 0.5-75 |
| S6-Methylphenidate | 16.5987 | 0.0514 | 0.9989 | 0.1 | 0.1-75 |
| S6-Nicotine | 6.1800 | -0.1768 | 0.9924 | 1 | 1-75 |
| S6-Nikethamide | 52.1893 | -1.1701 | 0.9987 | 0.5 | 0.5-75 |
| S6-Strychnine | 1.5180 | 0.0055 | 0.9979 | 0.5 | 0.5-75 |
| S6-Synephrine | / | / | / | / | / |
| S6-Phenylephrine | / | / | / | / | / |
| S7-Buprenorphine | 1.8409 | -0.0369 | 0.9996 | 0.05 | 0.05-3.75 |
| S7-Fentanyl | 2.1733 | 0.0208 | 0.9989 | 0.01 | 0.01-1.5 |
| S7-Heroin | 7.1127 | -0.0830 | 0.9984 | 0.25 | 0.25-37.5 |
| S7-Hydromorphone | 5.9584 | -0.1402 | 0.9995 | 1.25 | 1.25-37.5 |
| S7-Methadone | 0.9829 | -0.0021 | 0.9999 | 0.25 | 0.25-37.5 |
| S7-Morphine^2^ | 8.8692 | -0.1075 | 0.9999 | 4 | 4-300 |
| S7-Oxycodone | 27.8624 | -0.5645 | 0.9988 | 0.25 | 0.25-37.5 |
| S7-Oxymorphone | 4.7772 | -0.2430 | 0.9944 | 1.25 | 1.25-37.5 |
| S8-JWH-073 | 1.4815 | -0.0471 | 0.9966 | 0.05 | 0.05-1.5 |
| S9-Budesonide | 0.3263 | 0.0071 | 0.9994 | 2.25 | 2.25-67.5 |
| S9-Dexamethasone | 0.0278 | 0.0003 | 0.9964 | 1.2 | 1.2-90 |
| S9-Methylprednisolone | 0.0280 | -0.0009 | 0.9958 | 6 | 6-45 |
| S9-Prednisone | 4.0915 | 0.1529 | 0.9995 | 15 | 15-450 |

^1^The unit is ng/mL; ^2^For morphine, the MRPL is 1000 ng/mL, but for making the calibration curves, 200 ng/mL was used.

**Table S6.** The recovery of the CBS-MS and the sequential LC-MS method.

| Compound | CBS-MS | | | Sequential LC-MS | | |
| --- | --- | --- | --- | --- | --- | --- |
|  | 25% MPRL | 50% MPRL | 100% MRPL | 25% MPRL | 50% MPRL | 100% MRPL |
| P1-Acebutolol | 99 | 97 | 98 | 103 | 91 | 114 |
| P1-Alprenolol | 102 | 108 | 103 | 111 | 90 | 85 |
| P1-Atenolol | / | 108 | 100 | 98 | 98 | 101 |
| P1-Carvedilol | 101 | 93 | 96 | 102 | 99 | 81 |
| P1-Labetalol | 111 | 110 | 104 | 111 | 113 | 95 |
| P1-Metoprolol | 101 | 100 | 98 | 104 | 100 | 104 |
| P1-Nadolol | 100 | 99 | 99 | 97 | 94 | 97 |
| P1-Propranolol | 98 | 102 | 100 | 106 | 99 | 109 |
| P1-Timolol | 101 | 103 | 100 | 99 | 100 | 93 |
| S1-Clenbuterol | / | / | / | 93 | 106 | 104 |
| S1-Stanozolol | / | 105 | 102 | 91 | 94 | 97 |
| S1-Zilpaterol | / | 103 | 96 | 95 | 97 | 95 |
| S2-Desidustat | / | / | / | / | 89 | 84 |
| S3-Fenoterol | 97 | 95 | 95 | 96 | 104 | 96 |
| S3-Olodaterol | 102 | 102 | 101 | 107 | 103 | 92 |
| S3-Reproterol | / | 106 | 94 | 93 | 93 | 102 |
| S3-Salmeterol | 108 | 97 | 98 | 87 | 112 | 87 |
| S4-Anastrozole | / | / | / | / | 85 | 81 |
| S4-Arimistane | / | / | / | 73 | 78 | 104 |
| S4-Raloxifene | 92 | 89 | 92 | 86 | 97 | 72 |
| S4-Trimetazidine | 97 | 97 | 100 | 102 | 93 | 102 |
| S5-Acetazolamide | / | / | / | / | / | / |
| S5-Bumetanide | / | 109 | 117 | 99 | 104 | 78 |
| S5-Indapamide | 102 | 106 | 102 | 116 | 89 | 79 |
| S5-Probenecid | 93 | 107 | 97 | 89 | 89 | 79 |
| S5-Pseudoephedrine | 94 | 100 | 95 | 100 | 104 | 99 |
| S6-Cocaine | 103 | 102 | 100 | 116 | 99 | 102 |
| S6-Dimethylamphetamine | 84 | 94 | 95 | 85 | 91 | 90 |
| S6-Fenproporex | 96 | 95 | 93 | 91 | 98 | 93 |
| S6-MDMA | 97 | 100 | 98 | 100 | 98 | 94 |
| S6-Mefenorex | 97 | 106 | 100 | 108 | 101 | 84 |
| S6-Phentermine | 93 | 101 | 95 | 69 | 96 | 97 |
| S6-Ortetamine |  |  |  | 94 | 109 | 100 |
| S6-p-Methylamphetamine |  |  |  | 96 | 107 | 95 |
| S6-Methylphenidate | 96 | 107 | 104 | 109 | 100 | 81 |
| S6-Nicotine | 96 | 98 | 94 | 97 | 98 | 98 |
| S6-Nikethamide | 94 | 102 | 100 | 87 | 92 | 91 |
| S6-Strychnine | 99 | 92 | 91 | 98 | 98 | 103 |
| S6-Synephrine | / | / | / | / | / | / |
| S6-Phenylephrine |  |  |  | / | / | / |
| S7-Buprenorphine | 95 | 94 | 97 | 89 | 99 | 98 |
| S7-Fentanyl | 95 | 99 | 100 | 103 | 104 | 104 |
| S7-Heroin | 96 | 98 | 92 | 102 | 90 | 101 |
| S7-Hydromorphone | 100 | 99 | 105 | 93 | 93 | 100 |
| S7-Methadone | 99 | 99 | 96 | 101 | 100 | 99 |
| S7-Morphine | 98 | 98 | 101 | 99 | 98 | 98 |
| S7-Oxycodone | 96 | 98 | 97 | 99 | 96 | 96 |
| S7-Oxymorphone | 106 | 106 | 106 | 99 | 91 | 100 |
| S8-JWH-073 | / | 107 | 100 | 77 | 101 | 76 |
| S9-Budesonide | 96 | 102 | 96 | 93 | 94 | 108 |
| S9-Dexamethasone | 124 | 107 | 104 | 100 | 77 | 87 |
| S9-Methylprednisolone | / | / | / | 101 | 100 | 104 |
| S9-Prednisone | / | 124 | 104 | 96 | 115 | 84 |

**Table S7.** The reproducibility of the CBS-MS and the sequential LC-MS method.

| Compound | CBS-MS | | Sequential LC-MS | |
| --- | --- | --- | --- | --- |
|  | Intra-day (n=7) | Inter-day (n=3) | Intra-day (n=7) | Inter-day (n=3) |
| P1-Acebutolol | 4 | 0 | 2 | 12 |
| P1-Alprenolol | 6 | 2 | 13 | 2 |
| P1-Atenolol | 13 | 2 | 2 | 2 |
| P1-Carvedilol | 3 | 5 | 20 | 17 |
| P1-Labetalol | 4 | 7 | 9 | 5 |
| P1-Metoprolol | 4 | 2 | 1 | 2 |
| P1-Nadolol | 4 | 2 | 3 | 3 |
| P1-Propranolol | 4 | 3 | 13 | 4 |
| P1-Timolol | 5 | 2 | 1 | 5 |
| S1-Clenbuterol | / | / | 4 | 7 |
| S1-Stanozolol | 5 | 2 | 3 | 3 |
| S1-Zilpaterol | 10 | 5 | 2 | 7 |
| S2-Desidustat | / | / | 21 | 16 |
| S3-Fenoterol | 4 | 3 | 1 | 3 |
| S3-Olodaterol | 3 | 2 | 1 | 3 |
| S3-Reproterol | 8 | 6 | 4 | 19 |
| S3-Salmeterol | 6 | 9 | 6 | 11 |
| S4-Anastrozole | / | / | 15 | 15 |
| S4-Arimistane | 7 | 4 | 12 | 9 |
| S4-Raloxifene | 6 | 11 | 15 | 18 |
| S4-Trimetazidine | 3 | 1 | 2 | 3 |
| S5-Acetazolamide | / | / | / | / |
| S5-Bumetanide | 10 | 13 | 16 | 12 |
| S5-Indapamide | 11 | 5 | 8 | 16 |
| S5-Probenecid | 13 | 3 | 14 | 4 |
| S5-Pseudoephedrine | 8 | 16 | 3 | 7 |
| S6-Cocaine | 3 | 2 | 3 | 8 |
| S6-Dimethylamphetamine | 7 | 9 | 1 | 11 |
| S6-Fenproporex | 5 | 2 | 2 | 1 |
| S6-MDMA | 5 | 2 | 1 | 4 |
| S6-Mefenorex | 7 | 4 | 2 | 7 |
| S6-Phentermine | 4 | 6 | 5 | 5 |
| S6-Ortetamine |  |  | 1 | 10 |
| S6-p-Methylamphetamine |  |  | 1 | 7 |
| S6-Methylphenidate | 6 | 6 | 3 | 7 |
| S6-Nicotine | 5 | 4 | 1 | 3 |
| S6-Nikethamide | 8 | 2 | 3 | 5 |
| S6-Strychnine | 3 | 5 | 1 | 4 |
| S6-Synephrine | / | / | / | / |
| S6-Phenylephrine |  |  | / | / |
| S7-Buprenorphine | 11 | 5 | 1 | 10 |
| S7-Fentanyl | 1 | 4 | 6 | 4 |
| S7-Heroin | 7 | 1 | 1 | 4 |
| S7-Hydromorphone | 4 | 1 | 1 | 4 |
| S7-Methadone | 2 | 1 | 3 | 2 |
| S7-Morphine | 5 | 2 | 1 | 1 |
| S7-Oxycodone | 6 | 1 | 1 | 3 |
| S7-Oxymorphone | 4 | 5 | 1 | 4 |
| S8-JWH-073 | 9 | 7 | 23 | 4 |
| S9-Budesonide | 18 | 5 | 11 | 8 |
| S9-Dexamethasone | 18 | 10 | 15 | 4 |
| S9-Methylprednisolone | 11 | 8 | 12 | 13 |
| S9-Prednisone | 15 | 11 | 17 | 9 |

**Table S8.** The storage stability of the prohibited substances in desorption solution and on blade for 12 h at 20˚C.

| Compound | Sequential LC-MS (12 h in desorption solution) | | Sequential LC-MS (12 h on blade) | | |
| --- | --- | --- | --- | --- | --- |
|  | Ratio (%) | RSD (%, n=5) | | Ratio (%) | RSD (%, n=5) |
| P1-Acebutolol | 98 | 3 | | 100 | 4 |
| P1-Alprenolol | 103 | 4 | | 102 | 2 |
| P1-Atenolol | 100 | 8 | | 100 | 5 |
| P1-Carvedilol | 101 | 5 | | 100 | 3 |
| P1-Labetalol | 104 | 3 | | 103 | 2 |
| P1-Metoprolol | 100 | 1 | | 100 | 4 |
| P1-Nadolol | 106 | 2 | | 102 | 2 |
| P1-Propranolol | 100 | 6 | | 102 | 10 |
| P1-Timolol | 101 | 4 | | 99 | 3 |
| S1-Clenbuterol | 95 | 10 | | 87 | 12 |
| S1-Stanozolol | 113 | 5 | | 112 | 5 |
| S1-Zilpaterol | 111 | 14 | | 104 | 10 |
| S2-Desidustat | 77 | 22 | | 61 | 32 |
| S3-Fenoterol | 101 | 5 | | 99 | 3 |
| S3-Olodaterol | 101 | 1 | | 98 | 1 |
| S3-Reproterol | 104 | 7 | | 114 | 8 |
| S3-Salmeterol | 102 | 3 | | 102 | 3 |
| S4-Anastrozole | 107 | 24 | | 87 | 14 |
| S4-Arimistane | 90 | 5 | | 93 | 3 |
| S4-Raloxifene | 96 | 7 | | 106 | 12 |
| S4-Trimetazidine | 90 | 3 | | 98 | 8 |
| S5-Acetazolamide | / | / | | / | / |
| S5-Bumetanide | 110 | 1 | | 108 | 3 |
| S5-Indapamide | 92 | 4 | | 89 | 2 |
| S5-Probenecid | 101 | 6 | | 103 | 10 |
| S5-Pseudoephedrine | 92 | 2 | | 106 | 4 |
| S6-Cocaine | 98 | 7 | | 101 | 1 |
| S6-Dimethylamphetamine | 106 | 3 | | 105 | 2 |
| S6-Fenproporex | 103 | 3 | | 104 | 2 |
| S6-MDMA | 99 | 3 | | 100 | 2 |
| S6-Mefenorex | 97 | 4 | | 95 | 1 |
| S6-Phentermine | 90 | 7 | | 89 | 8 |
| S6-Ortetamine | 101 | 4 | | 100 | 2 |
| S6-p-Methylamphetamine | 103 | 2 | | 99 | 4 |
| S6-Methylphenidate | 99 | 3 | | 96 | 1 |
| S6-Nicotine | 103 | 6 | | 98 | 6 |
| S6-Nikethamide | 103 | 4 | | 97 | 4 |
| S6-Strychnine | 103 | 2 | | 100 | 2 |
| S6-Synephrine | / | / | | / | / |
| S6-Phenylephrine | / | / | | / | / |
| S7-Buprenorphine | 94 | 6 | | 92 | 4 |
| S7-Fentanyl | 103 | 4 | | 98 | 4 |
| S7-Heroin | 98 | 4 | | 101 | 4 |
| S7-Hydromorphone | 102 | 10 | | 102 | 6 |
| S7-Methadone | 102 | 1 | | 101 | 3 |
| S7-Morphine | 101 | 4 | | 106 | 6 |
| S7-Oxycodone | 100 | 5 | | 103 | 6 |
| S7-Oxymorphone | 106 | 7 | | 106 | 5 |
| S8-JWH-073 | 106 | 4 | | 104 | 4 |
| S9-Budesonide | 76 | 7 | | 82 | 12 |
| S9-Dexamethasone | 100 | 4 | | 98 | 4 |
| S9-Methylprednisolone | 100 | 4 | | 95 | 4 |
| S9-Prednisone | 98 | 6 | | 94 | 3 |

**Table S9.** The selectivity testing results for 12 urine samples.

| Compound | Blank 1 | Blank  2 | Blank 3 | Blank 4 | Blank 5 | Blank 6 | Blank 7 | Blank 8 | Blank 9 | Blank 10 | Blank 11 | Blank 12 |
| --- | --- | --- | --- | --- | --- | --- | --- | --- | --- | --- | --- | --- |
| P1-Acebutolol | 0.0 | 0.0 | 0.0 | 0.0 | 0.0 | 0.0 | 0.0 | 0.0 | 0.0 | 0.0 | 0.0 | 0.0 |
| P1-Alprenolol | 0.0 | 0.0 | 0.0 | 0.0 | 0.0 | 0.0 | 0.0 | 0.0 | 0.0 | 0.0 | 0.0 | 0.0 |
| P1-Atenolol | 0.0 | 0.0 | 0.1 | 0.0 | 0.1 | 0.1 | 0.1 | 0.1 | 0.0 | 0.0 | 0.0 | 0.0 |
| P1-Carvedilol | 0.0 | 0.0 | 0.0 | 0.0 | 0.0 | 0.0 | 0.0 | 0.0 | 0.0 | 0.0 | 0.0 | 0.0 |
| P1-Labetalol | 0.0 | 0.0 | 0.0 | 0.0 | 0.0 | 0.0 | 0.0 | 0.0 | 0.0 | 0.1 | 0.0 | 0.0 |
| P1-Metoprolol | 0.0 | 0.0 | 0.0 | 0.0 | 0.0 | 0.0 | 0.0 | 0.0 | 0.0 | 0.0 | 0.0 | 0.0 |
| P1-Nadolol | 0.0 | 0.0 | 0.0 | 0.0 | 0.0 | 0.0 | 0.0 | 0.0 | 0.0 | 0.0 | 0.0 | 0.0 |
| P1-Propranolol | 0.0 | 0.0 | 0.0 | 0.0 | 0.0 | 0.0 | 0.0 | 0.0 | 0.0 | 0.0 | 0.0 | 0.0 |
| P1-Timolol | 0.0 | 0.0 | 0.0 | 0.0 | 0.0 | 0.0 | 0.0 | 0.0 | 0.0 | 0.0 | 0.0 | 0.0 |
| S1-Clenbuterol | 0.1 | 0.1 | 0.1 | 0.1 | 0.1 | 0.2 | 0.2 | 0.1 | 0.6 | 0.4 | 0.6 | 0.4 |
| S1-Stanozolol | 0.2 | 0.2 | 0.2 | 0.1 | 0.1 | 0.1 | 0.1 | 0.1 | 0.3 | 0.2 | 0.3 | 0.3 |
| S1-Zilpaterol | 0.0 | 0.0 | 0.1 | 0.1 | 0.1 | 0.1 | 0.0 | 0.0 | 0.1 | 0.1 | 0.1 | 0.1 |
| S2-Desidustat | 0.7 | 0.3 | 0.5 | 0.5 | 0.7 | 0.6 | 1.0 | 0.6 | 1.9 | 2.1 | 1.6 | 1.5 |
| S3-Fenoterol | 0.0 | 0.0 | 0.0 | 0.0 | 0.0 | 0.0 | 0.0 | 0.0 | 0.0 | 0.0 | 0.0 | 0.0 |
| S3-Olodaterol | 0.0 | 0.0 | 0.0 | 0.0 | 0.0 | 0.0 | 0.0 | 0.0 | 0.0 | 0.0 | 0.0 | 0.0 |
| S3-Reproterol | 0.0 | 0.1 | 0.0 | 0.1 | 0.1 | 0.1 | 0.1 | 0.0 | 0.0 | 0.1 | 0.1 | 0.1 |
| S3-Salmeterol | 0.0 | 0.0 | 0.0 | 0.0 | 0.0 | 0.0 | 0.0 | 0.0 | 0.0 | 0.0 | 0.0 | 0.0 |
| S4-Anastrozole | 0.6 | 0.6 | 0.5 | 0.6 | 0.8 | 0.8 | 0.9 | 0.7 | 3.4 | 2.4 | 3.3 | 2.6 |
| S4-Arimistane | 0.3 | 0.3 | 0.2 | 0.2 | 0.2 | 0.2 | 0.3 | 0.3 | 0.6 | 0.5 | 0.7 | 0.6 |
| S4-Raloxifene | 0.0 | 0.0 | 0.0 | 0.0 | 0.0 | 0.0 | 0.0 | 0.0 | 0.0 | 0.0 | 0.0 | 0.0 |
| S4-Trimetazidine | 0.0 | 0.0 | 0.0 | 0.0 | 0.0 | 0.0 | 0.0 | 0.0 | 0.0 | 0.0 | 0.0 | 0.0 |
| S5-Acetazolamide | / | / | / | / | / | / | / | / | / | / | / | / |
| S5-Bumetanide | 0.1 | 0.2 | 0.1 | 0.0 | 0.1 | 0.0 | 0.1 | 0.1 | 0.2 | 0.1 | 0.3 | 0.0 |
| S5-Indapamide | 0.0 | 0.0 | 0.0 | 0.0 | 0.0 | 0.0 | 0.0 | 0.0 | 0.0 | 0.0 | 0.0 | 0.0 |
| S5-Probenecid | 0.0 | 0.0 | 0.0 | 0.0 | 0.0 | 0.1 | 0.1 | 0.0 | 0.1 | 0.1 | 0.1 | 0.2 |
| S5-Pseudoephedrine | 0.0 | 0.0 | 0.0 | 0.0 | 0.0 | 0.0 | 0.0 | 0.0 | 0.0 | 0.0 | 0.0 | 0.0 |
| S6-Cocaine | 0.0 | 0.0 | 0.0 | 0.0 | 0.0 | 0.0 | 0.0 | 0.0 | 0.0 | 0.0 | 0.0 | 0.0 |
| S6-Dimethylamphetamine | 0.0 | 0.0 | 0.0 | 0.0 | 0.0 | 0.0 | 0.0 | 0.0 | 0.0 | 0.0 | 0.0 | 0.0 |
| S6-Fenproporex | 0.0 | 0.0 | 0.0 | 0.0 | 0.0 | 0.0 | 0.0 | 0.0 | 0.0 | 0.0 | 0.0 | 0.0 |
| S6-MDMA | 0.0 | 0.0 | 0.0 | 0.0 | 0.0 | 0.0 | 0.0 | 0.0 | 0.0 | 0.0 | 0.0 | 0.0 |
| S6-Mefenorex | 0.0 | 0.0 | 0.0 | 0.0 | 0.0 | 0.0 | 0.0 | 0.0 | 0.0 | 0.0 | 0.0 | 0.0 |
| S6-Phentermine | 0.0 | 0.0 | 0.0 | 0.0 | 0.0 | 0.0 | 0.0 | 0.0 | 0.0 | 0.0 | 0.0 | 0.0 |
| S6-Ortetamine |  |  |  |  |  |  |  |  |  |  |  |  |
| S6-p-Methylamphetamine |  |  |  |  |  |  |  |  |  |  |  |  |
| S6-Methylphenidate | 0.0 | 0.0 | 0.0 | 0.0 | 0.0 | 0.0 | 0.0 | 0.0 | 0.0 | 0.0 | 0.0 | 0.0 |
| S6-Nicotine | 0.0 | 0.0 | 0.0 | 0.0 | 0.0 | 0.0 | 0.0 | 0.0 | 0.0 | 0.0 | 0.0 | 0.0 |
| S6-Nikethamide | 0.0 | 0.0 | 0.0 | 0.0 | 0.0 | 0.0 | 0.0 | 0.0 | 0.0 | 0.0 | 0.0 | 0.0 |
| S6-Strychnine | 0.0 | 0.0 | 0.0 | 0.0 | 0.0 | 0.0 | 0.0 | 0.0 | 0.0 | 0.0 | 0.0 | 0.0 |
| S6-Synephrine | / | / | / | / | / | / | / | / | / | / | / | / |
| S6-Phenylephrine |  |  |  |  |  |  |  |  |  |  |  |  |
| S7-Buprenorphine | 0.0 | 0.0 | 0.0 | 0.0 | 0.0 | 0.0 | 0.0 | 0.0 | 0.0 | 0.0 | 0.0 | 0.0 |
| S7-Fentanyl | 0.0 | 0.0 | 0.0 | 0.0 | 0.0 | 0.0 | 0.0 | 0.0 | 0.0 | 0.0 | 0.0 | 0.0 |
| S7-Heroin | 0.0 | 0.0 | 0.0 | 0.0 | 0.0 | 0.0 | 0.0 | 0.0 | 0.0 | 0.0 | 0.0 | 0.0 |
| S7-Hydromorphone | 0.0 | 0.0 | 0.0 | 0.0 | 0.0 | 0.0 | 0.0 | 0.0 | 0.0 | 0.0 | 0.0 | 0.0 |
| S7-Methadone | 0.0 | 0.0 | 0.0 | 0.0 | 0.0 | 0.0 | 0.0 | 0.0 | 0.0 | 0.0 | 0.0 | 0.0 |
| S7-Morphine | 0.0 | 0.0 | 0.0 | 0.0 | 0.0 | 0.0 | 0.0 | 0.0 | 0.0 | 0.0 | 0.0 | 0.0 |
| S7-Oxycodone | 0.0 | 0.0 | 0.0 | 0.0 | 0.0 | 0.0 | 0.0 | 0.0 | 0.0 | 0.0 | 0.0 | 0.0 |
| S7-Oxymorphone | 0.0 | 0.0 | 0.0 | 0.0 | 0.0 | 0.0 | 0.0 | 0.0 | 0.0 | 0.0 | 0.0 | 0.1 |
| S8-JWH-073 | 0.0 | 0.0 | 0.0 | 0.0 | 0.0 | 0.0 | 0.1 | 0.0 | 0.2 | 0.1 | 0.2 | 0.1 |
| S9-Budesonide | 0.1 | 0.0 | 0.1 | 0.0 | 0.2 | 0.1 | 0.1 | 0.0 | 0.2 | 0.1 | 0.1 | 0.3 |
| S9-Dexamethasone | 0.0 | 0.0 | 0.0 | 0.0 | 0.0 | 0.0 | 0.0 | 0.0 | 0.1 | 0.1 | 0.0 | 0.1 |
| S9-Methylprednisolone | 0.4 | 0.3 | 0.3 | 0.4 | 0.5 | 0.7 | 0.4 | 0.5 | 0.9 | 1.0 | 1.3 | 1.3 |
| S9-Prednisone | 0.1 | 0.2 | 0.1 | 0.1 | 0.1 | 0.1 | 0.1 | 0.1 | 0.4 | 0.5 | 0.5 | 0.4 |

**Table S10.** The reliability testing results for 12 urine samples.

| Compound | MRPL 1 | MRPL 2 | MRPL 3 | MRPL 4 | MRPL 5 | MRPL 6 | MRPL 7 | MRPL 8 | MRPL 9 | MRPL 10 | MRPL 11 | MRPL 12 |
| --- | --- | --- | --- | --- | --- | --- | --- | --- | --- | --- | --- | --- |
| P1-Acebutolol | 1.9 | 1.9 | 1.8 | 2.0 | 1.9 | 1.8 | 1.9 | 1.9 | 1.8 | 1.8 | 1.9 | 1.9 |
| P1-Alprenolol | 1.8 | 2.0 | 2.3 | 2.1 | 2.4 | 2.7 | 2.6 | 2.6 | 2.0 | 2.2 | 2.0 | 2.4 |
| P1-Atenolol | 2.2 | 2.1 | 1.9 | 2.0 | 2.0 | 1.7 | 1.7 | 1.9 | 1.2 | 1.2 | 1.1 | 1.1 |
| P1-Carvedilol | 2.1 | 1.9 | 1.8 | 1.9 | 1.6 | 1.7 | 1.8 | 1.9 | 1.9 | 1.8 | 2.3 | 2.4 |
| P1-Labetalol | 1.9 | 1.9 | 2.1 | 2.1 | 2.2 | 2.1 | 2.3 | 2.1 | 2.0 | 2.0 | 2.1 | 2.5 |
| P1-Metoprolol | 1.9 | 2.0 | 1.9 | 1.9 | 1.9 | 1.9 | 1.9 | 2.0 | 1.8 | 1.7 | 1.9 | 1.8 |
| P1-Nadolol | 2.0 | 1.7 | 1.8 | 1.9 | 1.7 | 1.6 | 1.9 | 1.8 | 1.6 | 1.6 | 1.7 | 1.5 |
| P1-Propranolol | 1.8 | 1.8 | 1.8 | 1.8 | 1.8 | 1.8 | 1.9 | 1.8 | 1.8 | 2.0 | 2.0 | 1.8 |
| P1-Timolol | 1.8 | 1.8 | 1.7 | 1.8 | 1.8 | 1.7 | 1.8 | 1.9 | 1.7 | 1.6 | 1.8 | 1.7 |
| S1-Clenbuterol | 1.6 | 1.8 | 1.4 | 1.4 | 1.9 | 1.8 | 1.8 | 1.8 | 2.2 | 1.9 | 2.1 | 2.1 |
| S1-Stanozolol | 1.9 | 1.9 | 1.9 | 1.9 | 1.8 | 1.8 | 1.8 | 1.8 | 2.0 | 1.8 | 1.7 | 1.7 |
| S1-Zilpaterol | 1.3 | 1.7 | 1.5 | 1.5 | 1.4 | 1.4 | 1.5 | 1.9 | 1.5 | 1.1 | 1.5 | 1.3 |
| S2-Desidustat | 1.9 | 1.4 | 1.9 | 1.0 | 2.6 | 2.3 | 1.1 | 1.6 | 2.1 | 1.4 | 2.3 | 1.3 |
| S3-Fenoterol | 2.0 | 2.0 | 1.8 | 2.0 | 2.1 | 1.7 | 1.8 | 1.9 | 2.2 | 2.2 | 2.3 | 2.0 |
| S3-Olodaterol | 1.9 | 2.0 | 2.0 | 1.9 | 1.9 | 2.0 | 1.9 | 2.0 | 2.0 | 2.0 | 1.9 | 1.9 |
| S3-Reproterol | 2.2 | 2.2 | 2.1 | 2.4 | 2.4 | 2.4 | 2.5 | 3.1 | 1.5 | 1.5 | 1.8 | 1.5 |
| S3-Salmeterol | 2.6 | 1.9 | 1.6 | 1.8 | 1.5 | 1.6 | 1.7 | 1.7 | 1.6 | 1.7 | 1.9 | 1.7 |
| S4-Anastrozole | 0.9 | 1.0 | 1.0 | 1.0 | 1.3 | 1.6 | 1.8 | 1.8 | 3.3 | 4.2 | 3.7 | 4.1 |
| S4-Arimistane | 1.5 | 1.6 | 1.6 | 1.8 | 2.0 | 1.7 | 1.9 | 2.1 | 2.7 | 2.2 | 2.8 | 2.7 |
| S4-Raloxifene | 1.6 | 1.4 | 1.3 | 1.4 | 1.2 | 0.9 | 1.1 | 1.1 | 1.5 | 1.6 | 1.6 | 1.3 |
| S4-Trimetazidine | 2.0 | 2.0 | 2.0 | 2.0 | 2.2 | 2.3 | 2.1 | 2.2 | 2.0 | 2.0 | 1.9 | 2.1 |
| S5-Acetazolamide | / | / | / | / | / | / | / | / | / | / | / | / |
| S5-Bumetanide | 1.7 | 1.6 | 1.5 | 1.4 | 2.2 | 1.6 | 2.1 | 2.0 | 1.6 | 1.5 | 1.2 | 1.9 |
| S5-Indapamide | 1.7 | 1.6 | 2.2 | 2.1 | 2.4 | 2.0 | 2.4 | 2.2 | 1.7 | 2.0 | 1.8 | 2.2 |
| S5-Probenecid | 1.5 | 1.7 | 2.3 | 2.0 | 2.4 | 1.8 | 2.0 | 1.9 | 1.9 | 1.8 | 1.9 | 2.1 |
| S5-Pseudoephedrine | 2.3 | 2.7 | 2.5 | 2.4 | 3.0 | 2.5 | 2.3 | 2.2 | 2.1 | 2.3 | 1.9 | 2.1 |
| S6-Cocaine | 1.8 | 1.8 | 1.8 | 1.8 | 1.8 | 1.9 | 1.8 | 1.7 | 1.9 | 1.9 | 1.9 | 1.8 |
| S6-Dimethylamphetamine | 2.1 | 2.1 | 2.1 | 2.2 | 2.2 | 2.3 | 2.2 | 2.2 | 2.0 | 1.8 | 2.1 | 1.9 |
| S6-Fenproporex | 1.5 | 1.7 | 1.7 | 1.6 | 1.6 | 1.5 | 1.7 | 1.7 | 1.7 | 1.7 | 2.0 | 1.7 |
| S6-MDMA | 1.9 | 1.9 | 1.9 | 1.9 | 1.8 | 1.9 | 1.9 | 1.9 | 2.0 | 1.7 | 2.0 | 1.9 |
| S6-Mefenorex | 1.7 | 1.8 | 2.3 | 2.0 | 2.1 | 2.5 | 2.1 | 2.2 | 2.0 | 2.5 | 1.6 | 1.8 |
| S6-Phentermine | 1.8 | 2.5 | 2.4 | 2.2 | 2.2 | 2.1 | 1.9 | 2.0 | 2.3 | 2.6 | 2.2 | 2.3 |
| S6-Ortetamine |  |  |  |  |  |  |  |  |  |  |  |  |
| S6-p-Methylamphetamine |  |  |  |  |  |  |  |  |  |  |  |  |
| S6-Methylphenidate | 1.6 | 1.7 | 2.3 | 1.9 | 2.2 | 2.4 | 2.2 | 2.1 | 2.0 | 2.5 | 1.6 | 1.8 |
| S6-Nicotine | 1.8 | 2.0 | 1.9 | 1.9 | 1.9 | 1.9 | 1.9 | 1.9 | 1.8 | 1.8 | 1.8 | 1.7 |
| S6-Nikethamide | 2.1 | 1.9 | 2.0 | 2.0 | 1.8 | 1.5 | 1.5 | 1.6 | 1.4 | 1.5 | 1.6 | 1.5 |
| S6-Strychnine | 1.8 | 1.6 | 1.5 | 1.6 | 1.4 | 1.4 | 1.6 | 1.5 | 1.6 | 1.5 | 1.7 | 1.5 |
| S6-Synephrine | / | / | / | / | / | / | / | / | / | / | / | / |
| S6-Phenylephrine |  |  |  |  |  |  |  |  |  |  |  |  |
| S7-Buprenorphine | 1.6 | 1.6 | 1.6 | 1.6 | 1.7 | 1.9 | 2.1 | 1.6 | 1.7 | 1.7 | 1.4 | 1.7 |
| S7-Fentanyl | 1.9 | 2.0 | 2.0 | 2.1 | 2.1 | 2.1 | 2.2 | 2.2 | 2.0 | 2.1 | 2.0 | 1.9 |
| S7-Heroin | 2.0 | 1.9 | 1.9 | 2.0 | 1.8 | 1.8 | 1.9 | 2.0 | 1.9 | 1.6 | 1.8 | 1.9 |
| S7-Hydromorphone | 2.0 | 2.0 | 1.8 | 1.9 | 2.1 | 2.0 | 2.0 | 1.9 | 1.5 | 1.9 | 1.5 | 2.0 |
| S7-Methadone | 1.8 | 1.9 | 1.7 | 1.8 | 1.8 | 1.8 | 1.9 | 1.8 | 1.8 | 1.8 | 1.8 | 1.8 |
| S7-Morphine | 1.9 | 2.0 | 1.8 | 1.9 | 2.0 | 2.1 | 2.1 | 1.9 | 1.6 | 2.0 | 2.0 | 1.4 |
| S7-Oxycodone | 1.9 | 1.8 | 1.9 | 1.7 | 1.7 | 1.8 | 1.9 | 2.1 | 1.8 | 1.7 | 1.8 | 1.6 |
| S7-Oxymorphone | 1.9 | 2.0 | 1.7 | 1.9 | 2.0 | 1.8 | 1.9 | 1.9 | 1.5 | 1.8 | 1.5 | 1.9 |
| S8-JWH-073 | 2.4 | 1.9 | 2.3 | 2.1 | 2.0 | 2.1 | 2.0 | 2.5 | 2.1 | 1.9 | 2.5 | 3.4 |
| S9-Budesonide | 1.9 | 2.1 | 2.0 | 1.9 | 1.9 | 2.0 | 1.8 | 2.2 | 2.2 | 1.7 | 1.8 | 2.4 |
| S9-Dexamethasone | 1.8 | 1.6 | 1.6 | 1.8 | 1.8 | 1.4 | 1.9 | 2.0 | 1.4 | 1.5 | 1.4 | 1.7 |
| S9-Methylprednisolone | 1.1 | 1.2 | 1.5 | 1.6 | 2.4 | 1.9 | 2.2 | 2.1 | 2.2 | 2.1 | 1.9 | 2.0 |
| S9-Prednisone | 1.6 | 1.5 | 2.2 | 1.6 | 2.6 | 2.2 | 2.7 | 1.9 | 1.6 | 2.5 | 1.6 | 1.9 |
